# Understanding the Thalidomide Chirality in Biological Processes by the Self-disproportionation of Enantiomers

**DOI:** 10.1038/s41598-018-35457-6

**Published:** 2018-11-20

**Authors:** Etsuko Tokunaga, Takeshi Yamamoto, Emi Ito, Norio Shibata

**Affiliations:** 1 Department of Nanopharmaceutical Sciences and Department of Life Science and Applied Chemistry, Nagoya Institute of Technology, Gokiso, Showa-ku, Nagoya, 466-8555 Japan; 2 Institute of Advanced Fluorine-Containing Materials, Zhejiang Normal University, 688 Yingbin Avenue, 321004 Jinhua, China

## Abstract

Twenty years after the thalidomide disaster in the late 1950s, Blaschke *et al*. reported that only the (*S*)-enantiomer of thalidomide is teratogenic. However, other work has shown that the enantiomers of thalidomide interconvert *in vivo*, which begs the question: why is teratogen activity not observed in animal experiments that use (*R*)-thalidomide given the ready *in vivo* racemization (“thalidomide paradox”)? Herein, we disclose a hypothesis to explain this “thalidomide paradox” through the *in-vivo* self-disproportionation of enantiomers. Upon stirring a 20% ee solution of thalidomide in a given solvent, significant enantiomeric enrichment of up to 98% ee was observed reproducibly in solution. We hypothesize that a fraction of thalidomide enantiomers epimerizes *in vivo*, followed by precipitation of racemic thalidomide in (*R*/*S*)-heterodimeric form. Thus, racemic thalidomide is most likely removed from biological processes upon racemic precipitation in (*R*/*S*)-heterodimeric form. On the other hand, enantiomerically pure thalidomide remains in solution, affording the observed biological experimental results: the (*S*)-enantiomer is teratogenic, while the (*R*)-enantiomer is not.

## Introduction

Thalidomide is one of the most notorious drugs, responsible for a tragic global medical disaster of limb malformations in the late 1950s^1–7^. Thalidomide had mainly been prescribed as a sleeping drug/sedative, but also to pregnant women for the relief of morning sickness. However, the increased number of limb malformations was later confirmed to be caused by thalidomide. Although thalidomide was banned in 1962 in Germany, and later worldwide, it has once again attracted clinical interest due to the discovery of its unique pharmacological action against a number of intractable diseases, such as leprosy, human immunodeficiency virus replication in acquired immune deficiency syndrome, and cancer^8–16^. Thalidomide is now on the market with the new brand name THALOMID® for the treatment of patients with diagnosed multiple myeloma and severe erythema nodosum leprosum.

**Figure 1:**
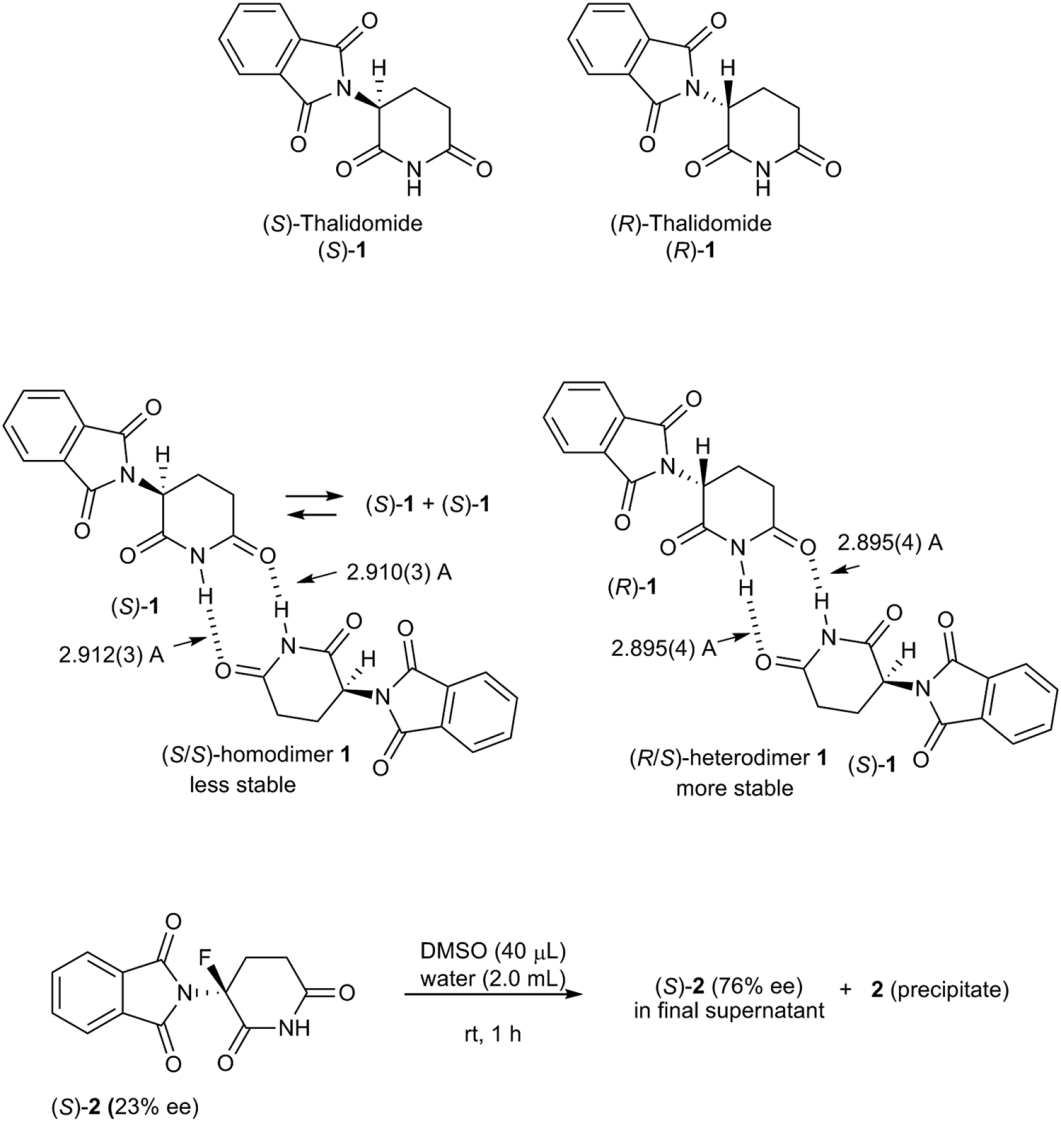
(*S*)- and (*R*)-enantiomers of thalidomide (**1**).

Thalidomide (**1**) possesses a single stereogenic carbon center and thus (*S*)- and (*R*)-enantiomers (Fig. 1). Commercially, it was marketed as a racemate. In 1979, Blaschke *et al*. discovered that the enantiomers of **1** display different biological properties and that only the (*S*)-enantiomer of **1** is responsible for the teratogenic side effects, while no teratogenicity was observed for (*R*)-**1** in animal experiments^17^. This paper suggested that the thalidomide disaster could have been avoided if only (*R*)-**1** had been marketed instead of racemic **1**. However, it is uncertain whether any of the actions of racemic thalidomide (**1**) could be avoided by using a pure enantiomer of **1**, due to the considerable racemization observed after incubation of enantiomerically pure **1** in buffer solution (τ_1/2_ = 12 h)^18,19^ as well as in serum (τ_1/2_ = 1 h)^20^. Thus, how can these results be rationalized in the light of the findings by Blaschke *et al*., which confirmed that (*R*)-**1** is not teratogenic despite the *in vivo* racemization? Although several enantioselective biological studies on the metabolism of thalidomide have been reported^21^, the confirmation of the different biological activity of the pure thalidomide enantiomers has ultimately remained problematic. In 2010, Handa and co-workers carried out a landmark biological study on thalidomide by identifying cereblon (CRBN) as a thalidomide-binding protein^22,23^. They found that thalidomide induces its teratogenic effects by binding to CRBN in zebrafish and chicks. This report opened a new era for thalidomide in science and completely redirected the research on this drug^24–27^. In 2018, Hakoshima, Handa, and co-workers, including two of the authors of the present study, finally put an end to the debate on the teratogenicity of (*S*)-thalidomide via structural and biochemical studies of the (*S*)- and (*R*)-enantiomers of thalidomide coordinated to CRBN^28^. The biochemical studies were carried out on deuterium-substituted enantiomers of thalidomide^29^ to suppress any enantiomeric interconversion. The results revealed that the (*S*)-enantiomer of **1** displayed a 10-fold stronger binding to CRBN and inhibition of self-ubiquitylation compared to the (*R*)-isomer. The crystal structure of the thalidomide-binding domain of CRBN bound to each of the enantiomers revealed that both bind to the CRBN pocket, although the bound form of the (*S*)-enantiomer exhibits a more natural ring conformation of the six-membered glutarimide moiety. Thus, the report by Blaschke is absolutely correct, i.e., the teratogenic effects are induced exclusively by the (*S*)-enantiomer of thalidomide.

However, one question remains regarding the clear biological differences between the thalidomide enantiomers reported in 1979, despite the observed racemization of thalidomide. Why do animal experiments that use (*R*)-**1** not display teratogenicity if (*R*)-**1** readily racemizes *in vivo*? This discrepancy is known as the “thalidomide paradox”. During our continuous studies on **1** and its derivatives^30–38^, we noticed that the physicochemical properties of racemic **1** and its pure enantiomers are very different^39^. Herein, we disclose a hypothesis to explain the “thalidomide paradox” based on the self-disproportionation of the enantiomers of non-racemic thalidomide in biological processes.

## Results

**Figure 2:**
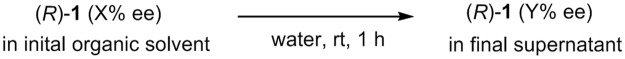
Self-disproportionation of non-racemic **1** (for Table 1).

The term self-disproportionation of enantiomers (SDE) was introduced by Soloshonok in 2006 to describe the transformation of an enantiomerically enriched system through the formation of fractions with a different proportion of enantiomers relative to the original ratio^40–46^. Our investigation began by examining whether SDE would occur in a solution of **1**. A solid mixture of (*R*)- and (*S*)-**1** (*R*/*S* = ~3 mg/2 mg) was dissolved in a variety of organic solvents, the enantiomeric excess (ee) was determined, and then water (1.0 mL) was added. In these solutions, the progressive formation of a precipitate was observed. After 1 h, the ee of **1** in the supernatant was determined by HPLC using a chiral column (Fig. 2, Table 1). In EtOH (8 mL), the initial ee of (*R*)-**1** was 21%, while the final supernatant showed 22% ee of (*R*)-**1**, (entry 1, Table 1). A similar result was obtained in CHCl_3_/EtOH (1:1, 4 mL, entry 2). Interestingly, the SDE of **1** was also observed in DMSO (entries 3–4). The use of 0.2 mL of DMSO as the organic solvent resulted in an increased enantiomeric excess of (*R*)-**1** (50% ee) in the supernatant relative to the initial value (16% ee, entry 3). After optimization of the volume, 25 μL of DMSO was found to be effective for this SDE: an initial 19% ee of (*R*)-**1** afforded enantioenriched (*R*)-**1** with 83% ee in the supernatant, while a substantial amount of precipitate was also observed (entry 4). Further details for the applied conditions are shown in the Supplementary Information (Table S1).

**Table 1:** Self-disproportionation of non-racemic **1**^a^.

Entry	X (% ee)^b^	Organic solvent	Y (% ee)^b^
1	21	EtOH (8 mL)	21
2	16	CHCl_3_/EtOH (2 mL/2 mL)	17
3^c^	16	DMSO (0.2 mL)	50
4^c^	19	DMSO (25 μL)	83

^a^The (*R*)-**1** sample was prepared by dissolving (*S*)-**1** (2 mg) and (*R*)-**1** (3 mg) in the organic solvent; the initial ee (X% ee) was determined directly at this point. Then, the SDE experiment was carried out upon addition of water (1 mL). The final ee (Y% ee) was determined from the supernatant after 1 h. ^b^The ee was determined by HPLC using a CHIRALCEL OJ-H column with ethanol as the eluent. ^c^In the final solution, a substantial amount of precipitate was observed.

**Figure 3:**
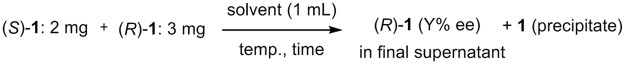
Self-disproportionation of non-racemic **1** in water and phosphate buffer (pH = 7) (for Table 2).

Next, experiments under conditions simulating biological media were carried out, i.e., without organic solvents. Solid (*R*)- and (*S*)-**1** (*R*/*S* = 3 mg/2 mg) were mixed and ground by pestle, before water (1 mL) was added (Fig. 3, Table 2). After vigorous stirring for 1 h at room temperature, the supernatant of the suspension contained 97% ee of (*R*)-**1** (entry 1, Table 2). High enantiomeric excess values for (*R*)-**1** (97% and 91% ee) were also obtained after stirring at 37 °C for 1 and 24 h, respectively (entries 2 and 3). The use of phosphate buffer (pH = 7) as the solvent at room temperature provided (*R*)-**1** with a similarly high enantiomeric excess (98% and 89% ee) independently of the stirring time (entries 4 and 5). At 37 °C in phosphate buffer, 98% ee of (*R*)-**1** was detected in the supernatant after 1 h, which decreased to 62% ee after 24 h of stirring (entries 6 and 7). This phenomenon was also observed for (*S*)-**1**, which provided enantioenriched (*S*)-**1** (89% ee) under the same conditions (entry 8). Other ratios of (*R*)- and (*S*)-**1** were also examined in water and similar behavior was observed (entries 9–11). In all cases, a substantial amount of precipitate was observed in the final solution, and the precipitate showed a low ee of **1** (*vide infra*). Further details for the different conditions applied are shown in the Supplementary Information (Table S2).

**Figure 4:**
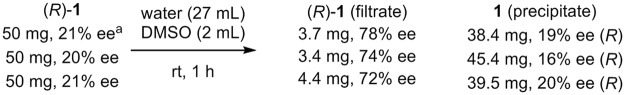
Self-disproportionation of non-racemic thalidomide at practical scale; ^a^DMSO (0.25 mL) was used.

Subsequently, we attempted to carry out the SDE of non-racemic thalidomide at a practical scale (Fig. 4). For that purpose, (*R*)-**1** (50 mg, 20% ee) was dissolved in DMSO (0.25 mL or 2 mL) before water (27 mL) was added. After stirring for 1 h at room temperature, the formed precipitate was filtered off. The filtrate and precipitate were dried separately by freeze drying. The filtrate afforded (*R*)-**1** (3.7 mg) with 78% ee, while the precipitate contained 19% ee of (*R*)-**1**. This experiment was repeated three times and similar results were obtained.

**Table 2:** Self-disproportionation of non-racemic **1** in water and phosphate buffer (pH = 7)^a^.

Entry	Solvent	Temp. (°C)	Time (h)	Y (% ee)^b^
1	Water	rt	1	97
2	Water	37	1	97
3	Water	37	24	91
4	Phosphate buffer	rt	1	98
5	Phosphate buffer	rt	24	88
6	Phosphate buffer	37	1	98
7	Phosphate buffer	37	24	62
8^c^	Water	rt	1	89 (*S*)^d^
9^e^ (17% ee)^f^	Water	rt	1	87
10^g^ (9.9% ee)^f^	Water	rt	1	74
11^h^ (4.8% ee)^f^	Water	rt	1	61

^a^All reactions were performed using a finely ground mixture of (*S*)-**1** (2 mg) and (*R*)-**1** (3 mg) in water (1 mL) or phosphate buffer (pH = 7; 1 mL), except entries 9–11. In all cases, a substantial amount of precipitate was observed in the final reaction mixture. ^b^The ee was determined by HPLC using a CHIRALCEL OJ-H column with ethanol as the eluent. ^c^The sample was prepared by mixing of (*R*)-**1** (2 mg) and (*S*)-**1** (3 mg), followed by addition of water (1 mL). ^d^(*S*)-**1** was obtained. ^e^A finely ground mixture of (*S*)-**1** (2.1 mg) and (*R*)-**1** (2.9 mg) was used. ^f^The initial ee was determined using a small amount of the ground mixture in DMSO prior to the addition of water. ^g^A finely ground mixture of (*S*)-**1** (2.3 mg) and (*R*)-**1** (2.7 mg) was used. ^h^A finely ground mixture of (*S*)-**1** (2.4 mg) and (*R*)-**1** (2.6 mg) was used.

Then, we examined the solubility of enantiomeric and racemic **1**. The solubility of **1** in water was determined by absorption spectroscopy. For that purpose, a saturated solution of **1** in water was prepared by stirring enantiomeric or racemic **1** (10 mg) in water (5 mL) at room temperature (23 °C) for 1 h. After filtering off all insoluble solids, the saturated water solution of **1** (1.0 mL) was diluted to 100 mL, before the UV spectrum was recorded. The water solubility of **1** was calculated using a calibration curve. The water solubility of (*S*)-**1** (348.9 ± 2.9 μg/mL) and (*R*)-**1** (344.9 ± 3.0 μg/mL) was found to be 5.5 times higher than that of racemate-**1** (62.5 ± 1.6 μg/mL).

**Figure 5:**
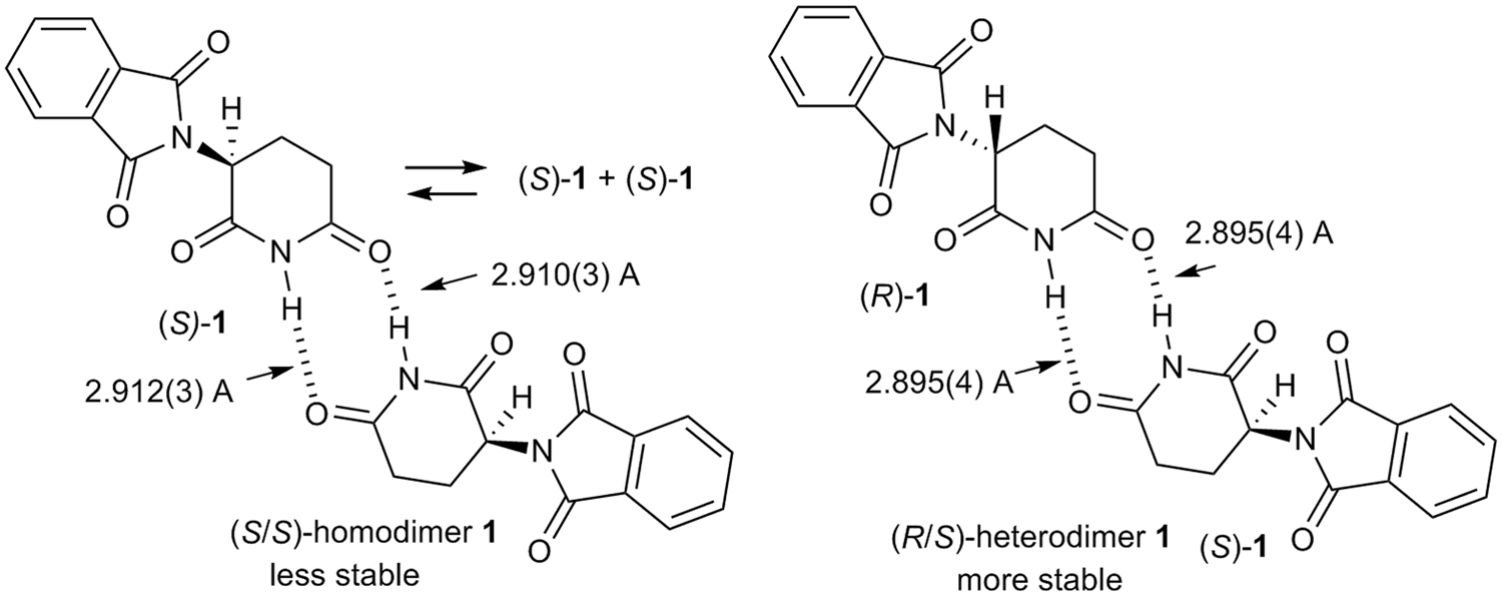
Structures of the (*S/S*)-homodimer and the (*R/S*)-heterodimer of **1** based on X-ray crystallographic analyses.

Such differences in the solubility of the individual enantiomers and the racemate of thalidomide **1** are in good agreement with the differences in their X-ray crystal structures that have previously been reported by us^39,47^. The crystal structure of (*S*)-**1** shows two types of arrangements: a solvated monomer and a non-solvated homodimer of two (*S*)-**1** molecules with hydrogen-bonding at the glutarimide rings. On the other hand, a non-solvated heterodimer of (*R*)- and (*S*)-thalidomide molecules was obtained for racemic-**1**. Obviously, the solvated monomer of (*S*)-**1** is more soluble than the other structures. Moreover, the homodimer of (*S*)-**1** should be more soluble than the heterodimer of racemic **1**, as the latter is more closely packed on account of two intermolecular hydrogen bonds. Namely, the lengths of two intermolecular hydrogen bonds of (*S/S*)-homodimer **1** are 2.912(3) Å and 2.910(3) Å, while those of racemic (*R*/*S*)-heterodimer **1** are 2.895(4) Å (Fig. 5).

## Discussion

**Figure 6:**
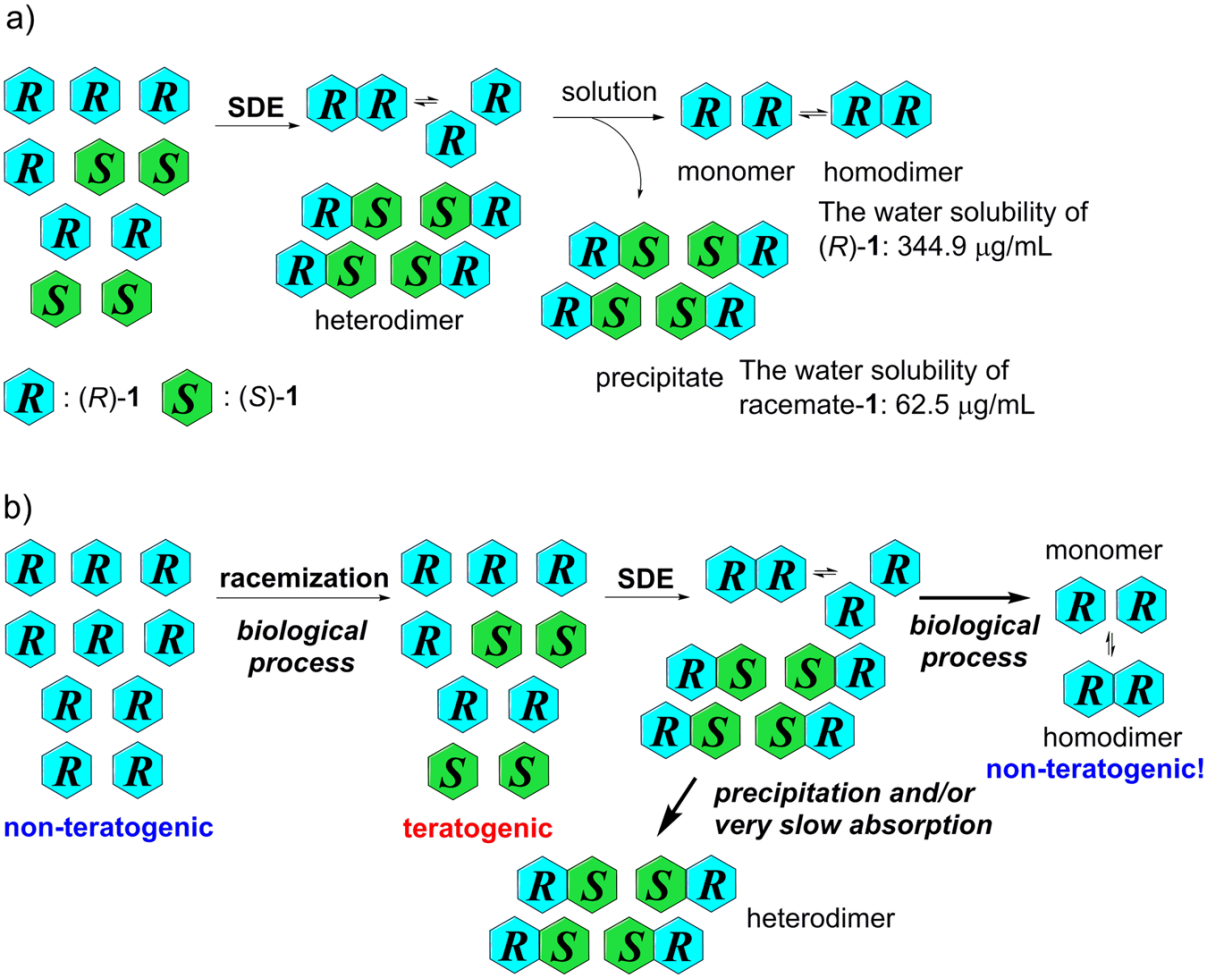
(**a**) Mechanism of the SDE for (*R*)-**1**. (**b**) An SDE-based hypothesis that feasibly explains why (*R*)-**1** does not show any teratogenic effect *in vivo*.

The SDE of non-racemic thalidomide in water (Tables 1 and 2 as well as Fig. 4) can be explained by the differences in the solubility of the enantiomers and the racemate of **1**. First, (*R*)-**1** in low ee gradually undergoes disproportionation into enantiomerically pure (*R*)-**1** (as a monomer and (*R*/*R*)-homodimer) and racemate-**1** (as an (*R*/*S*)-heterodimer). Due to the low solubility of the (*R*/*S*)-heterodimer, racemate-**1** precipitates, while enantiomerically enriched (*R*)-**1** remains in solution (Fig. 6a). The SDE of (*S*)-**1** can be explained in the same way. This phenomenon of racemic precipitation of the (*R*/*S*)-heterodimer could have some relations to asymmetric amplification reactions in which (*R*/*S*)-heterodimers have reduced catalytic activity relative to that of enantiomerically pure monomeric catalysts^48,49^.

The results by Blaschke *et al*.^17^ are thus most likely correct, provided that the SDE of thalidomide occurs in living biological systems. First, the acidic hydrogen atom at the asymmetric center of enantiomerically pure (*R*)-**1** epimerizes under physiological conditions, resulting in the formation of enantiomerically poor (*R*)-**1** (e.g. 20% ee). Such enantiomer mixtures of thalidomide with low ee spontaneously undergo a SDE to provide enantiopure (*R*)-**1** and racemic **1**. Racemic **1** is then removed from the biological medium by precipitation, while enantiopure (*R*)-**1** remains in solution to reach the target cells and/or organs (Fig. 6b). This hypothesis is also supported by the fact that the oral absorption of racemic thalidomide is slower than that of the individual thalidomide enantiomers^50,51^. Since the report by Blaschke *et al*. used large doses of thalidomide (<200 mg/kg for (*S*)-**1** and <400 mg/kg for (*R*)-**1**)^17^, this phenomenon is even more likely to occur.

**Figure 7:**
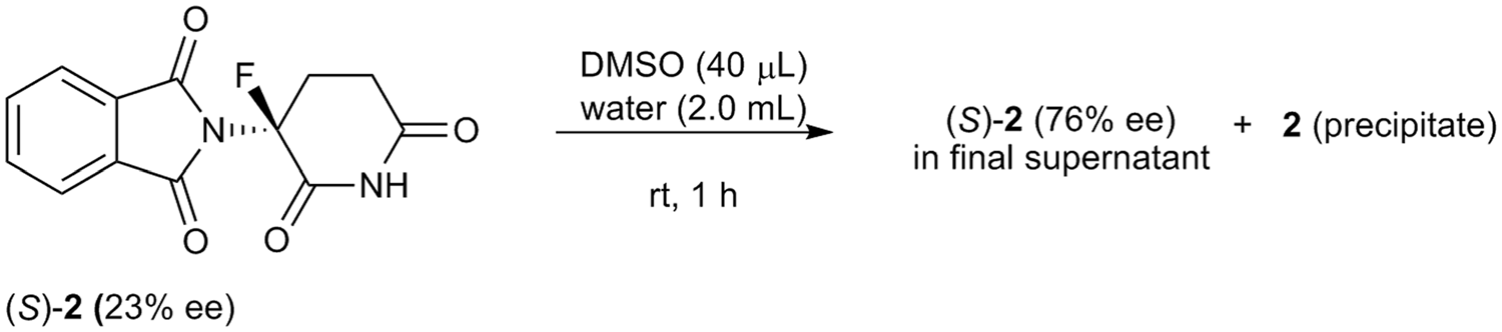
Self-disproportionation of non-racemic **2** in water. (*S*)-**2** (23% ee, 5 mg) was dissolved in DMSO, and the SDE experiment was carried out upon addition of water. The final ee was determined from the supernatant after 1 h of stirring by HPLC using a CHIRALCEL OJ-H column with ethanol as the eluent. A substantial amount of precipitate was also observed.

Finally, we carried out similar experiments using non-racemic fluoro-thalidomide **2**^30,37^, which is a non-racemized bioisostere of thalidomide **1**. Compound **2** exhibits potent anti-tumor activity against the human multiple myeloma cell line H929^38^, while it is non-teratogenic^36^. We thus briefly optimized the conditions for SDE of non-racemic **2** in water (*cf*. Table S3) and similar results were observed, i.e., (*S*)-**2** (23% ee) was initially dissolved in DMSO, before water was added. After vigorous stirring for 1 h at room temperature, the supernatant of the suspension contained <76% ee of (*S*)-**2**, while a substantial amount of precipitate was observed (Fig. 7).

In summary, we have disclosed the SDE of non-racemic thalidomide (**1**) in water and phosphate buffer solution. (*R*)-**1** (20% ee) undergoes SDE in water and aqueous buffer solution to furnish racemic **1** (as an (*R*/*S*)-heterodimer) in the form of a precipitate and enantiomerically enriched (*R*)-**1** (as the monomer and the (*R*/*R*)-homodimer) in solution (<98% ee). The same SDE was also observed for (*S*)-**1** (20% ee) in the same solvent system. The SDE of non-racemic **1** induces a spontaneous separation into dissolved and precipitating components based on the differences in solubility of the different structures of **1**, i.e., the monomer, the (*S*/*S*- or *R*/*R*)-homodimer of enantiomerically pure **1**, and the (*R*/*S*)-heterodimer of racemic **1**. The SDE, which is highly likely to occur in biological processes, provides a useful hypothesis to explain the “thalidomide paradox” for the first time since 1979. Since the SDE of non-racemic fluoro-thalidomide (**2**) was also observed, this phenomenon may also occur in other chiral drugs^52,53^. Thus, chiral drugs with low enantiomeric purity should be used only with extreme care due to potential SDE processes in biological systems.

## Materials and Methods

### Materials

(*R*)-**1**, (*S*)-**1**, and (*S*)-**2** were prepared according to previously reported methods^31,37^.

### SDE of non-racemic 1 in water

Water (1.0 mL) was added to a mixture of (*S*)-**1** (2.0 mg) and (*R*)-**1** (3.0 mg) in DMSO (25 μL) after the initial ee of the solution had been determined by HPLC using a CHIRALCEL OJ-H column with ethanol as the eluent. After stirring at room temperature for 1 h, the precipitate was filtered off and the enantiomeric excess of the filtrate was determined by HPLC.

### SDE of non-racemic 1 under simulated biological conditions

A mixture of (*S*)-**1** (2.0 mg), (*R*)-**1** (3.0 mg), and solvent (water or phosphate buffer, 1.0 mL) was stirred at room temperature or 37 °C for 1 or 24 h. The thus obtained precipitate was filtered off and the enantiomeric excess of the filtrate was determined by HPLC.

### SDE of non-racemic 1 on a practical scale

Water (27.0 mL) was added to a mixture of (*S*)-**1** (20.0 mg) and (*R*)-**1** (30.0 mg) in DMSO (0.25 mL) after the initial ee of the solution had been determined by HPLC. After stirring for 1 h at room temperature, the resulting precipitate was filtered off. The filtrate and precipitate were dried by freeze drying. (*R*)-**1** was detected both in the filtrate (3.8 mg, 38% yield, 84% ee) and the precipitate (43.0 mg, 15% ee (*R*)).

### Water solubility of the enantiomers and racemate of 1

Calibration curves were prepared by plotting the UV absorbance of aqueous solutions of **1** (0.5, 1.0, 2.5, 3.5 and 5.0 mg/L)^54^. A saturated solution of **1** in water was prepared by stirring enantiomeric or racemic **1** (10 mg) in water (5 mL) at room temperature for 1 h. After removing the resulting insoluble solid by filtration, the saturated aqueous solution (1.0 mL) was diluted to 100 mL and then the UV absorbance was measured. The solubility of **1** in water was calculated using the calibration curves.

## Electronic supplementary material

Supplementary Information
