# Supplementary material for: Understanding the Thalidomide Chirality in Biological Processes by the Self-disproportionation of Enantiomers
Source: Sci Rep. 2018 Nov 20;8:17131. doi: 10.1038/s41598-018-35457-6 (PMC6244226; doi:10.1038/s41598-018-35457-6)
Supplement: Supplementary file 1 — Supplementary Information [file 41598_2018_35457_MOESM1_ESM.pdf]

Supplementary Information

Understanding the Thalidomide Chirality in Biological Processes by the Self-disproportionation of Enantiomers

Etsuko Tokunaga,[1] Takeshi Yamamoto,[1] Emi Ito,[1] and Norio Shibata[1,2*]

[1]*Department of Nanopharmaceutical Sciences and Department of Life Science and Applied Chemistry, Nagoya Institute of Technology, Gokiso, Showa-ku, Nagoya 466-8555, Japan.*
[2]*Institute of Advanced Fluorine-Containing Materials, Zhejiang Normal University, 688 Yingbin Avenue, 321004 Jinhua, China*

Content

1. **Table S1 Self-disproportionation of non-racemic 1……………………………………………………………………..2**
2. **Table S2 Self-disproportionation of non-racemic (*R*)-1 in water and phosphate buffer (pH = 7)…..3**
3. **Table S3 Self-disproportionation of non-racemic (*S*)-2 in water……………………………………………….3**
4. **Water solubility of the enantiomers and racemate 1 and their calibration curves………………………4**
5. **Copies of ^{1}H NMR spectra of (*R*)-1……………………………………………………………………………………………6**
6. **Copies of ^{1}H and ^{19}F NMR spectra of (*S*)-2………………………………………………………………………………7**
7. **Copies of HPLC analysis of optically pure (*R*)-1 and (*S*)-1………………………………………………………………8**
8. **Copies of HPLC analysis of optically pure (*S*)-2 and (*R*)-2………………………………………………………………9**
9. **Copies of HPLC analysis of (*R*)-1 after self-disproportionation of non-racemic (*R*)-1 for Table 1, Table 2, and Fig. 4 ……………………………………………………………10**
10. **Copies of HPLC analysis of (*S*)-2 after self-disproportionation of non-racemic (*S*)-2, for Fig. 7 ....20**

Table S1 Self-disproportionation of non-racemic **1**

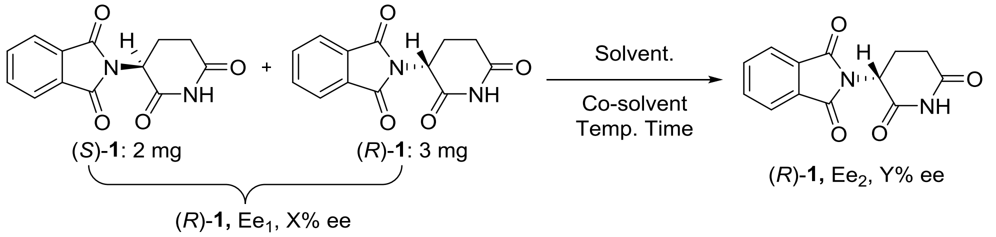

Entry	Solvent	Co-solvent	Temp. (°C)	Time (h)	Ee_1 (%)	Ee_2 (%)
1	water (1 mL)	DMSO (50 μL)	rt	1	21	74
2	water (1 mL)	DMSO (40 μL)	rt	1	34	85
3	water (1 mL)	DMSO (30 μL)	rt	1	19	80
4	water (1 mL)	DMSO (25 μL)	rt	1	20	83
5	water (1 mL)	DMSO (20 μL)	rt	1	–	80
6	water (1 mL)	DMSO (10 μL)	rt	1	–	85
7	water (1 mL)	DMSO (7 μL)	rt	1	–	89
8	water (1 mL)	DMSO (5 μL)	rt	1	–	80
9	water (1 mL)	DMSO (3 μL)	rt	1	–	94
10	water (2 mL)	DMSO (30 μL)	rt	1	34	81
11	water (2 mL)	DMSO (3 μL)	rt	1	–	98
12	water (0.5 mL)	DMSO (3 μL)	rt	1	–	81
13	phosphate buffer (pH 7) (2 mL)	DMSO (3 μL)	rt	1	–	98

The (*R*)-**1** sample was prepared by dissolving (*S*)-**1** (2 mg) and (*R*)-**1** (3 mg) in DMSO; the initial ee (Ee_1, X% ee) was determined directly at this point. Then, the SDE experiment was carried out upon addition of water (Z mL). The final ee (Ee_2, Y% ee) was determined from the supernatant after 1 h. The ee was determined by HPLC using a CHIRALCEL OJ-H column with ethanol as the eluent.

Table S2 Self-disproportionation of non-racemic **1** in water and phosphate buffer (pH = 7)

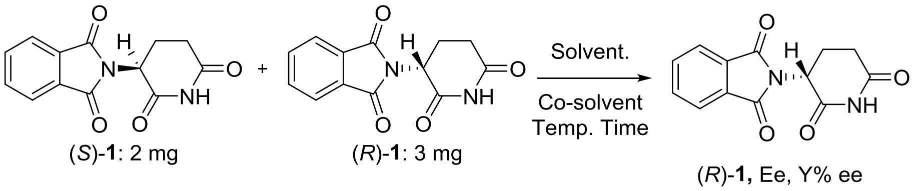

Entry	Solvent	Co-solvent	Temp. (°C)	Time (h)	Ee (%)
1	water (1 mL)	-	rt	1	97
2[a]	water (1 mL)	-	rt	1	98
3	water (1 mL)	-	rt	24	57
4	phosphate buffer (pH 7) (1 mL)	-	rt	1	98
5	phosphate buffer (pH 7) (1 mL)	-	rt	24	89
6	phosphate buffer (pH 7) (1 mL)	-	37	1	98
7	phosphate buffer (pH 7) (1 mL)	-	37	24	62
8	water (1 mL)	-	37	1	97
9	water (1 mL)	-	37	24	91

All reactions were performed using a finely ground mixture of (*S*)-**1** (2 mg) and (*R*)-**1** (3 mg) in solid-state and water (1 mL) or phosphate buffer (pH = 7; 1 mL). The ee was determined by HPLC using a CHIRALCEL OJ-H column with ethanol as the elutant.

[a]Solid (*R*)- and (*S*)-**1** (*R/S* = 3 mg/2 mg) were mixed together and directly water (1 mL) was added without grounding by pestle.

Table S3 Self-disproportionation of non-racemic (*S*)-2 in water.

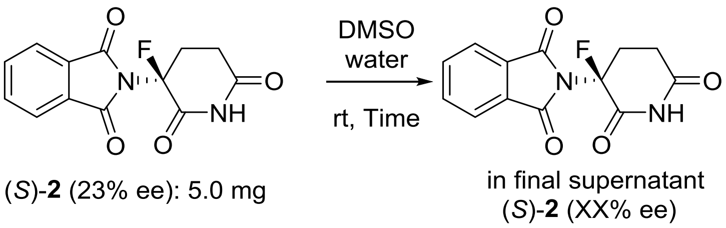

Entry	DMSO (μL)	water (mL)	Time (h)	Ee (%)
1	25	1	1	30.2
2	30	1	1	62.5
3	40	1	1	67.7
4	50	1	1	49.6
5	40	0.5	1	57.2
6	40	2.0	1	76.2
7	40	1.0	1	67.3
8	40	1.0	1	29.5
9	40	1.0	1	65.6
10	40	1.0	1	64.5
11	40	1.0	1	46.3

The (*S*)-**2** sample (5 mg, 23% ee) in DMSO was added into water (X mL). The final ee was determined from the supernatant after 1 h. The ee was determined by HPLC using a CHIRALCEL OJ-H column with ethanol as the eluent.

Water solubility of the enantiomers and racemate 1.

Calibration curves were prepared by plotting the UV absorbance of aqueous solutions of **1** (0.5, 1.0, 2.5, 3.5, and 5.0 mg/L). A saturated solution of **1** in water was prepared by stirring enantiomeric or racemic **1** (10 mg) in water (5 mL) at room temperature (23°C for 1 h. After removing the resulting insoluble solid by filtration, the saturated aqueous solution (1.0 mL) was diluted to 100 mL and then the UV absorbance was measured. The solubility of **1** in water was calculated using the calibration curves.
See reference; Hess, S.; Akermann, M. A.; Wnendt, S.; Zwingenberger, K.; Eger, K. *Bioorg. Med. Chem.* **2001**, *9*, 1279.

Results[a]

	Water solubility (µg/mL)	Relative water solubility to rac-**1**
rac-**1**	62.5 ± 1.6	1.00
(*S*)-**1**	348.9 ± 2.9	5.58
(*R*)-**1**	344.9 ± 3.0	5.52

[a]Water solubility was measured for three times. Date are shown as mean ± SD (n=3)

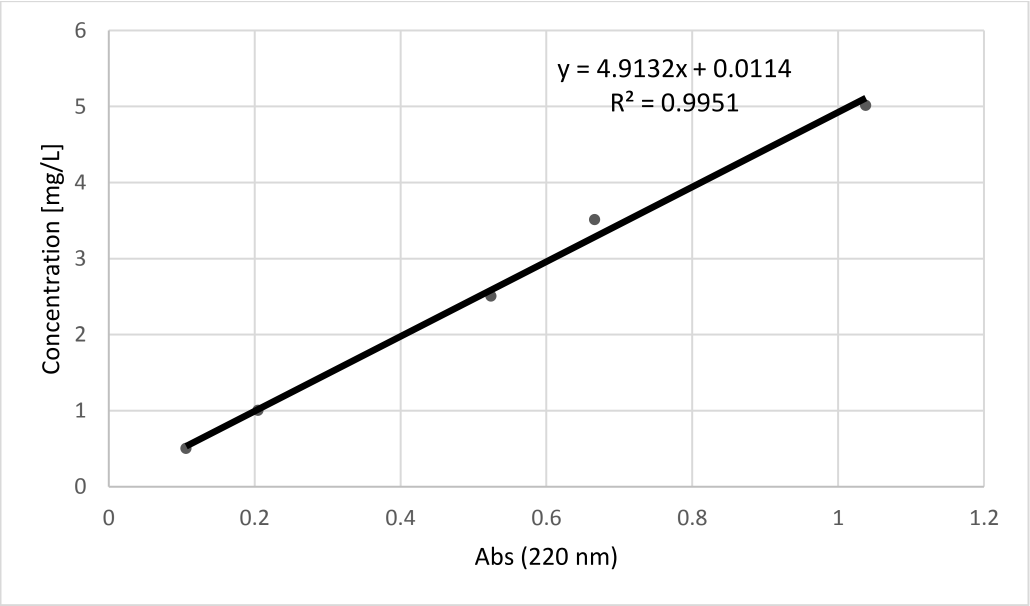

Calibration curve of Racemic **1**

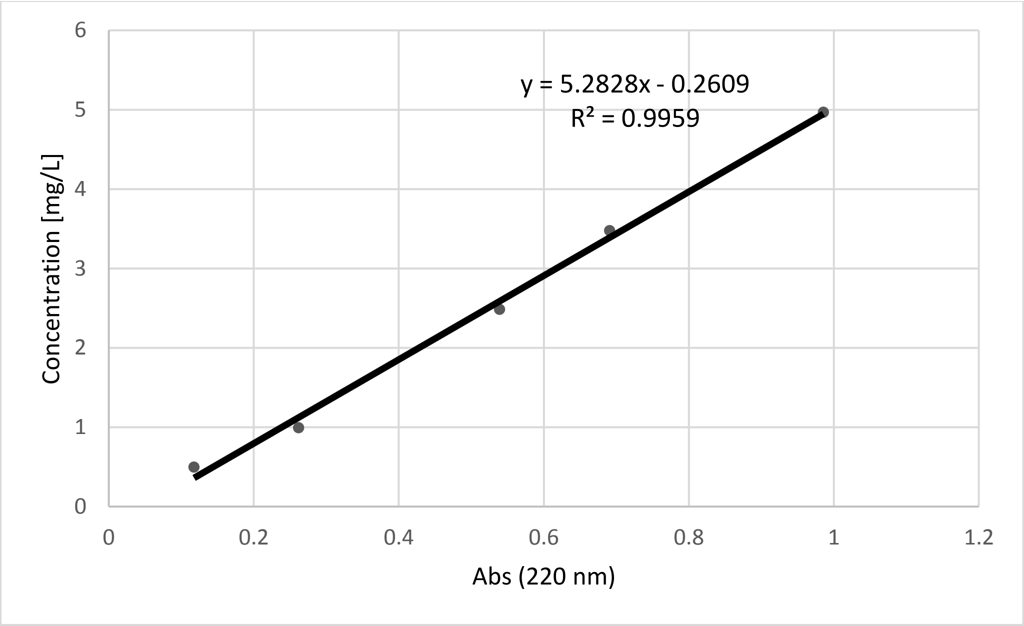

Calibration curve of (*S*)-**1**

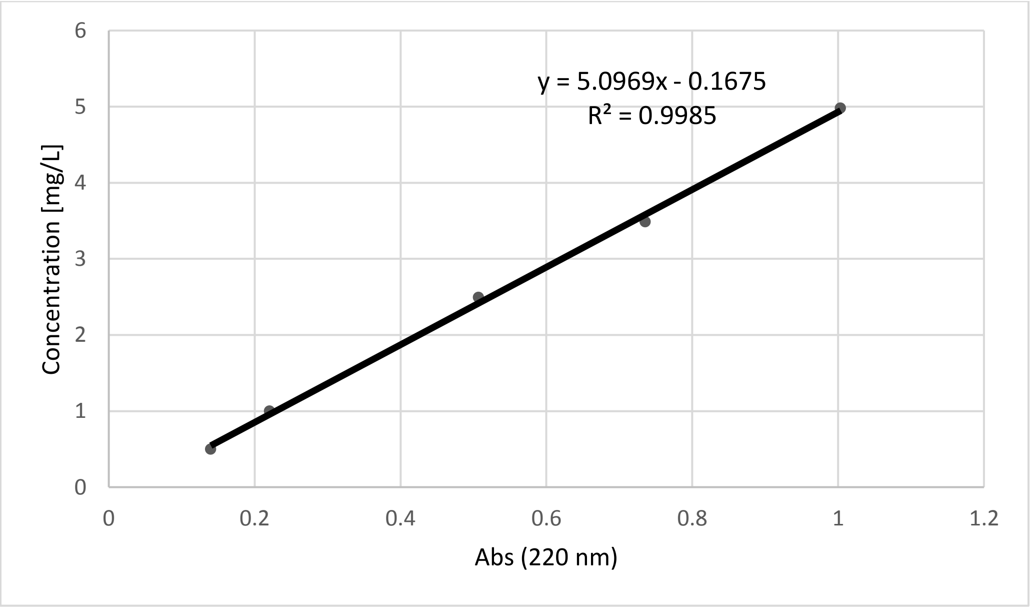

Calibration curve of (*R*)-**1**

^{1}H NMR of (*R*)-**1**

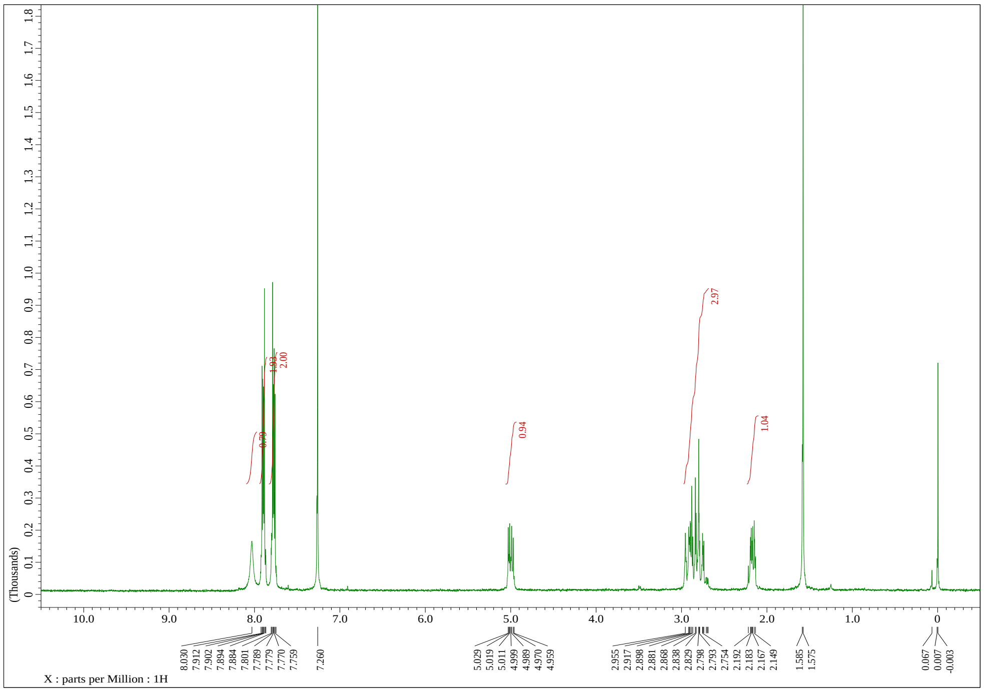

^{1}H-NMR (300 MHz, $CDCl_3$ δ 8.03 (s, 1H), 7.92–7.87 (m, 2H), 7.80–7.75 (m, 2H), 5.03–4.96 (m, 1H), 2.96–2.69 (m, 3H), 2.22–2.13 (m, 1H).

^{1}H NMR of (*S*)-**2**

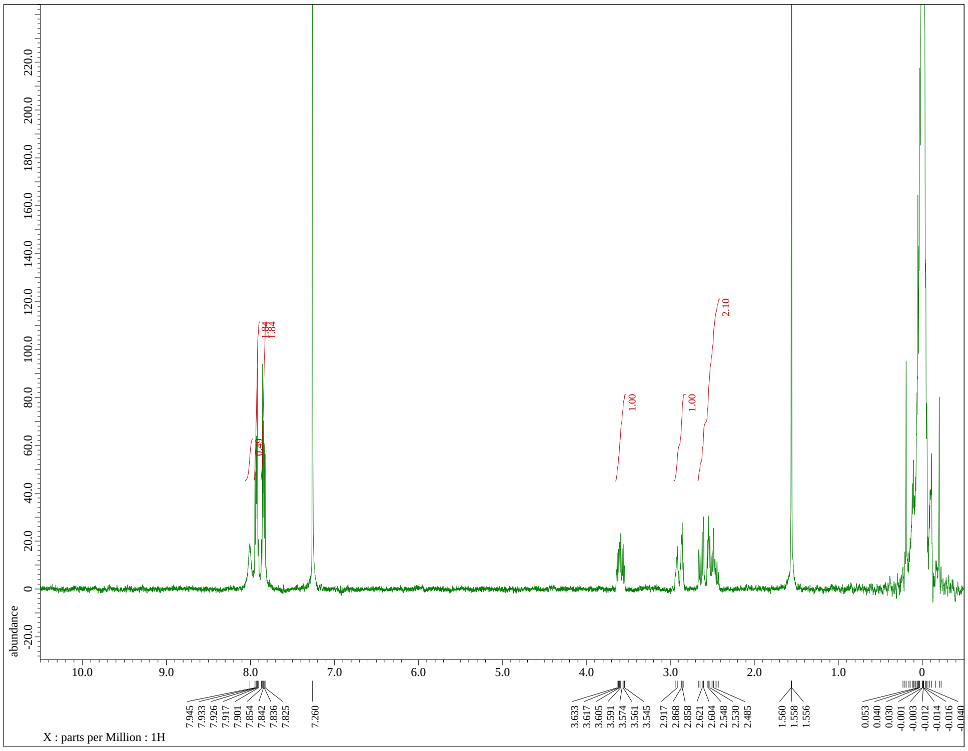

^{1}H-NMR (300 MHz, $CDCl_3$) δ 8.01 (s, 1H), 7.95–7.90 (m, 2H), 7.87–7.82 (m, 2H), 3.63–3.55 (m, 1H), 2.94–2.85 (m, 1H), 2.66–2.43 (m, 2H).

^{19}F NMR of (*S*)-**2**

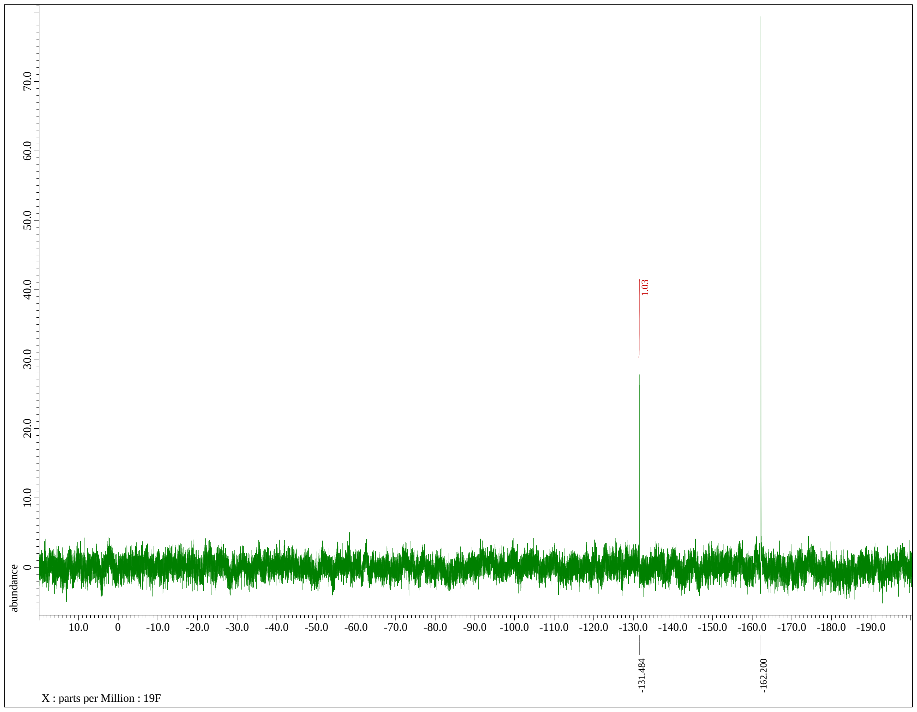

^{19}F NMR (282 MHz, $CDCl_3$) δ -131.5 (s, 1F)

HPLC of (*R*)-**1**

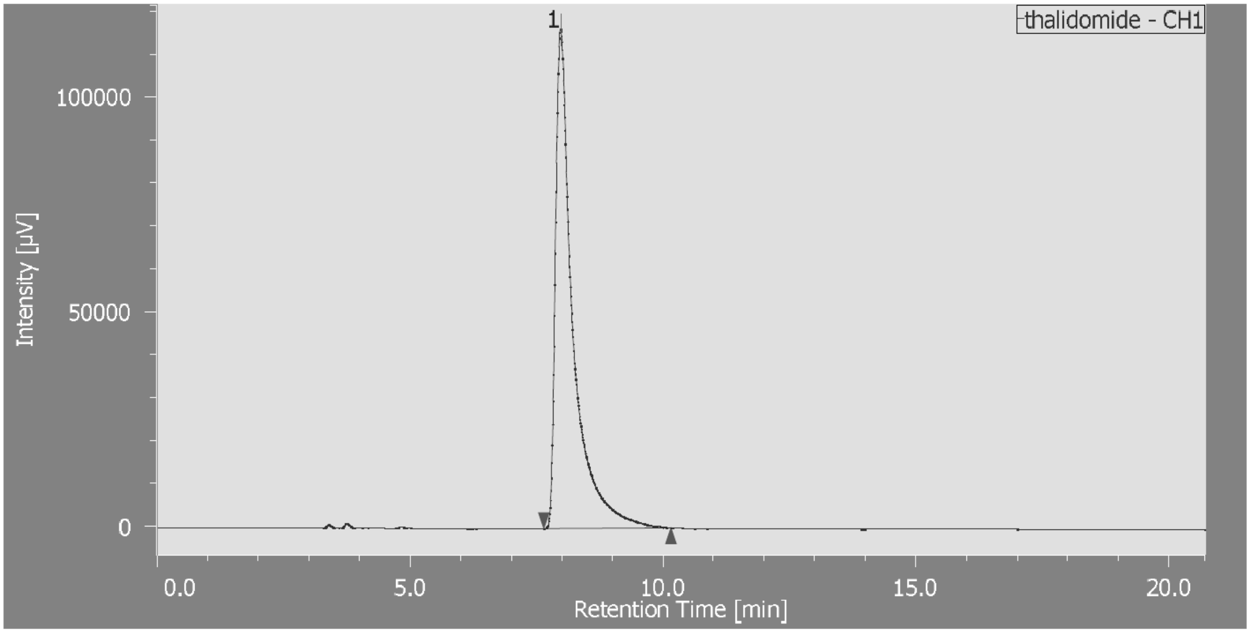

Peak 1: tR[min] 7.975; Area: 100%

HPLC of (*S*)-**1**

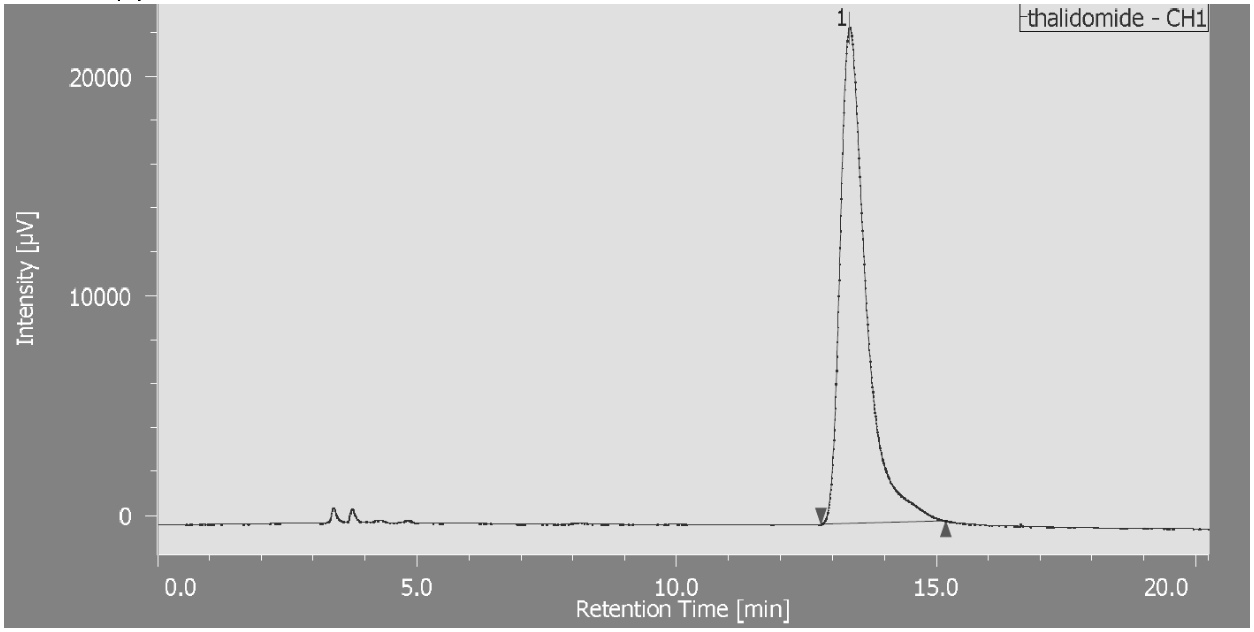

Peak 1: tR[min] 13.325; Area: 100%

HPLC of (*S*)-**2**

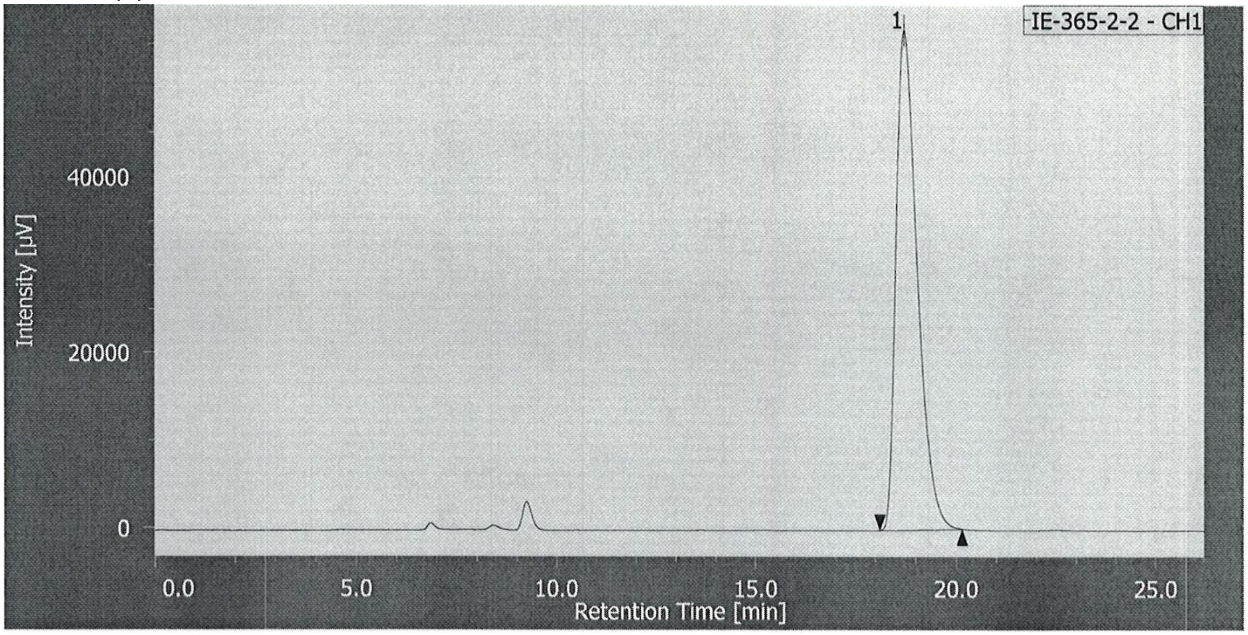

Peak 1: tR[min] 18.700; Area: 100%

HPLC of (*R*)-**2**

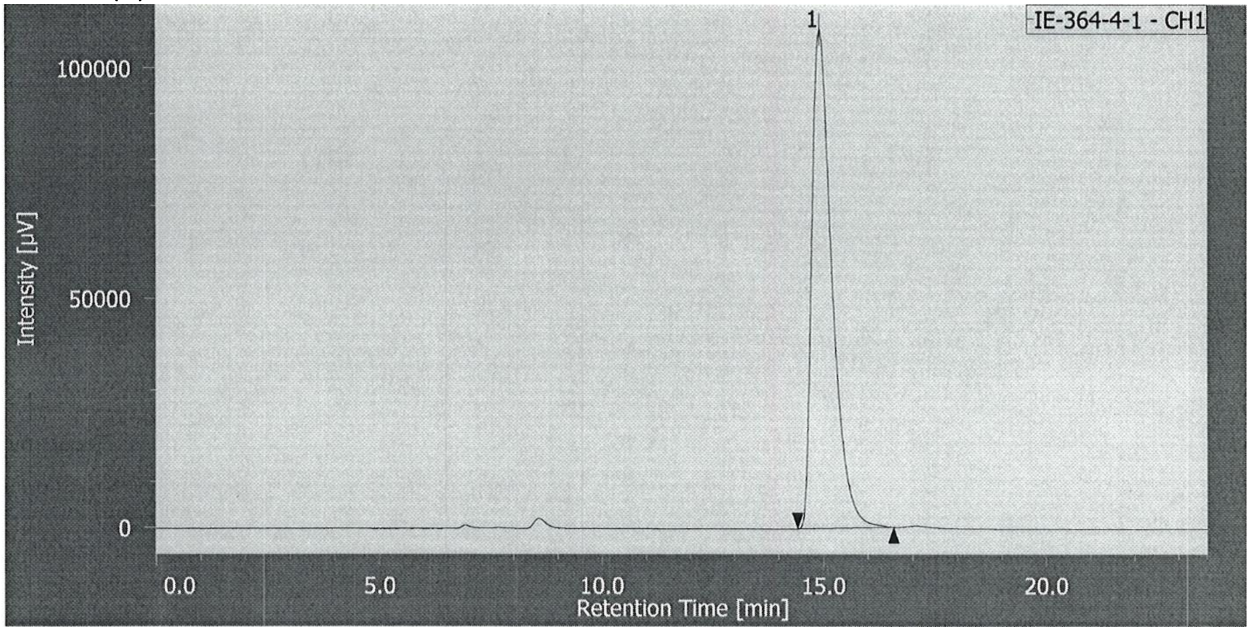

Peak 1: tR[min] 14.908; Area: 100%

Table 1, entry 3: 16% ee →50% ee, (*R*)-**1**

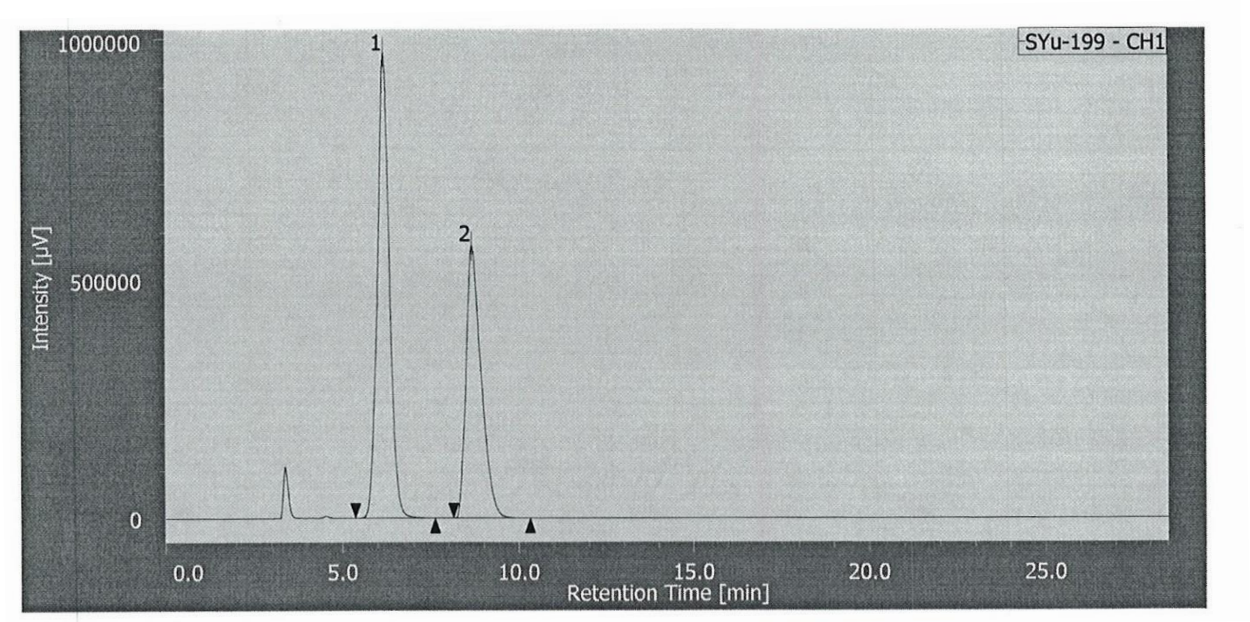

Peak 1: tR[min] 6.167; Area: 57.751%
Peak 2: tR[min] 8.658; Area: 42.249%

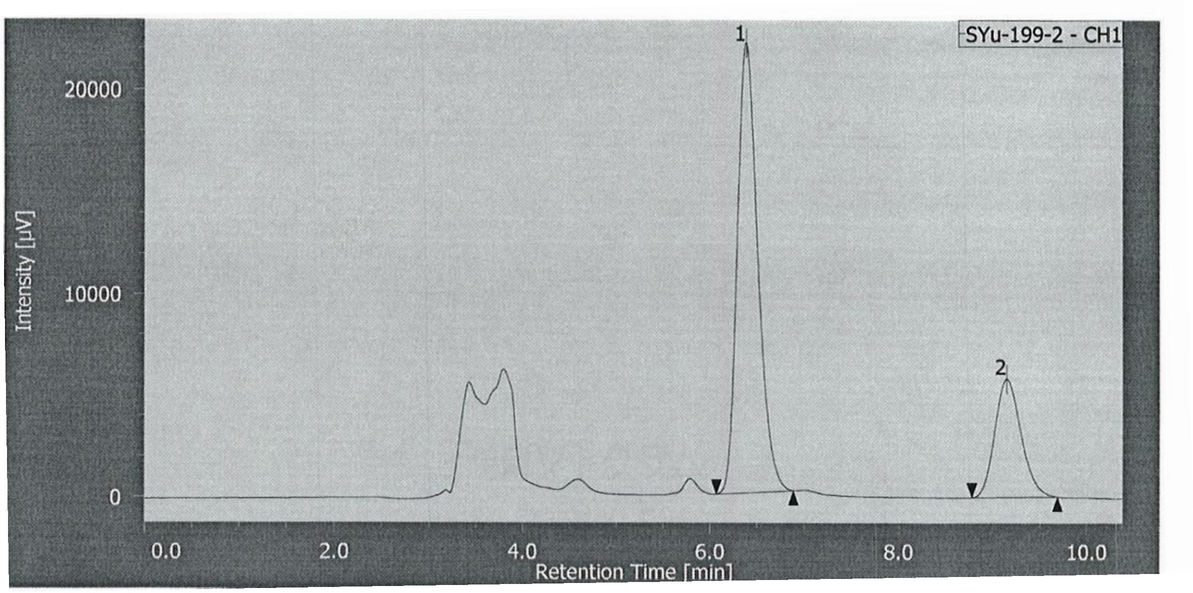

Peak 1: tR[min] 6.383; Area: 74.796%
Peak 2: tR[min] 9.142; Area: 25.204%

Table 1, entry 4: 19% ee → 83% ee, (*R*)-**1**

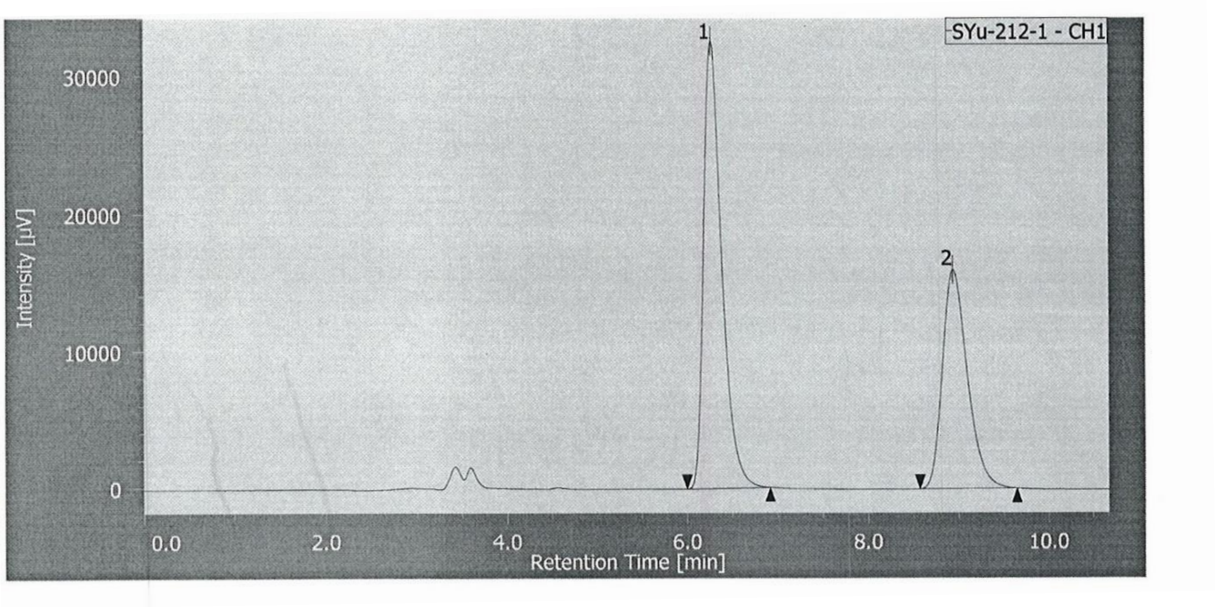

Peak 1: tR[min] 6.267; Area: 59.661%
Peak 2: tR[min] 8.933; Area: 40.339%

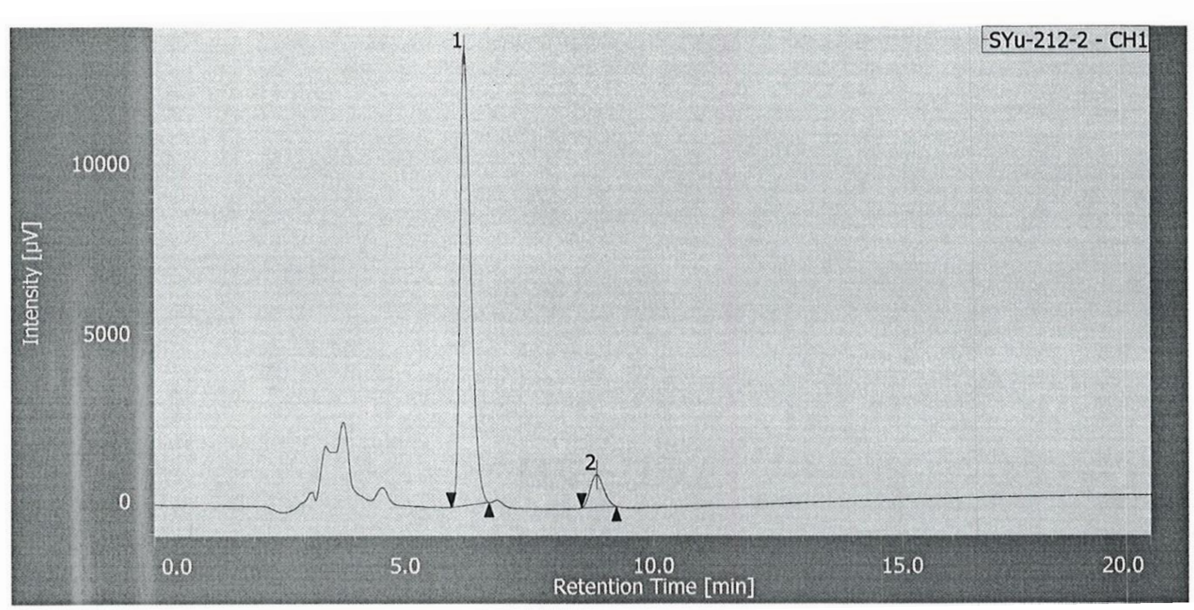

Peak 1: tR[min] 6.200; Area: 91.698%
Peak 2: tR[min] 8.850; Area: 8.302%

Table 2 entry 1 (water, rt, 1 h 97% ee), (*R*)-**1**

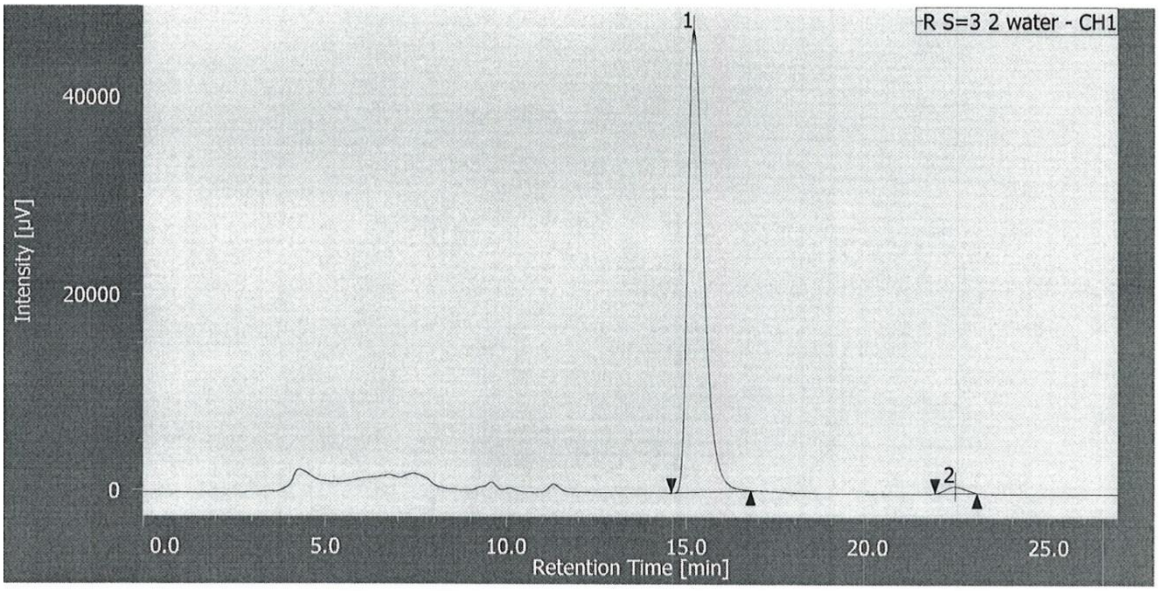

Peak 1: tR[min] 15.200; Area: 98.310%
Peak 2: tR[min] 22.408; Area: 1.690%

Table 2 entry 2 (water, 37 °C, 1 h 97% ee), (*R*)-**1**

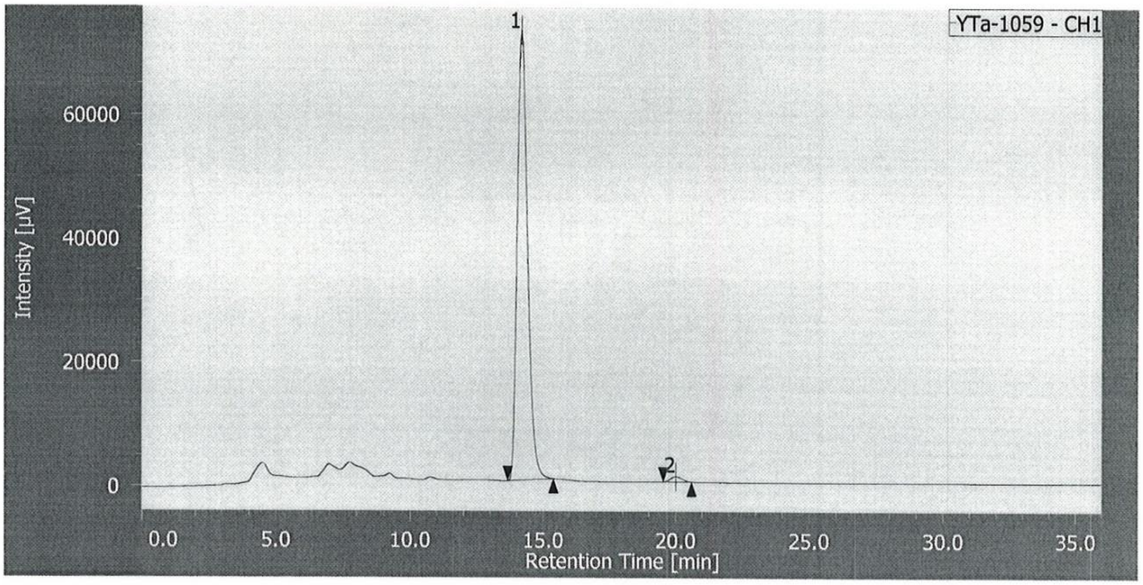

Peak 1: tR[min] 14.158; Area: 98.696%
Peak 2: tR[min] 20.008; Area: 1.304%

Table 2 entry 3 (water, 37 °C, 24 h 91% ee), (*R*)-**1**

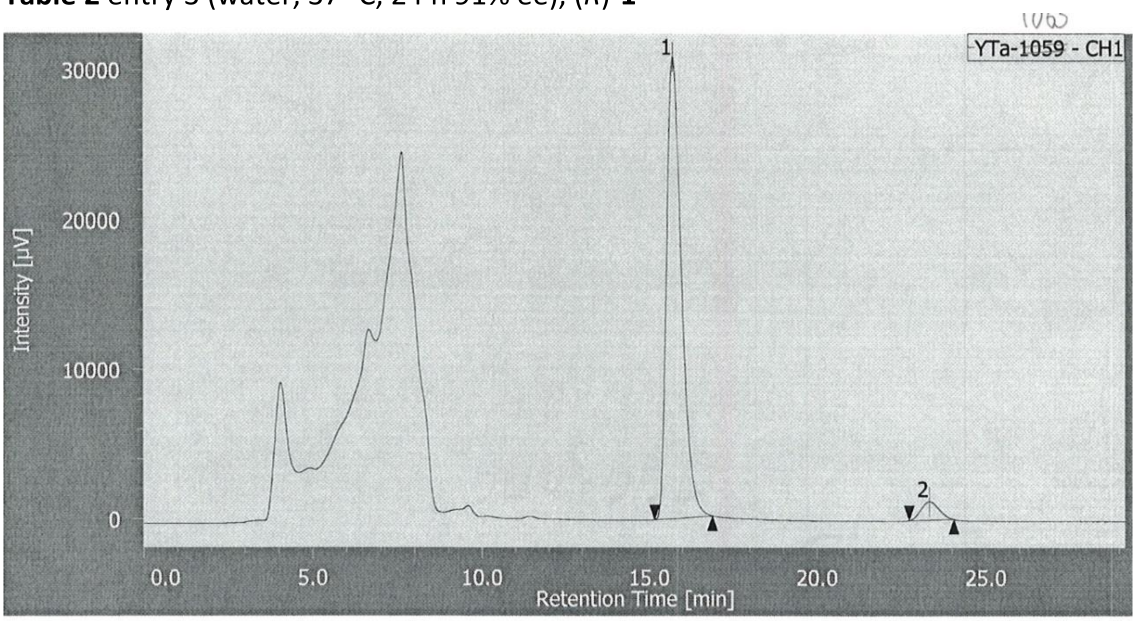

Peak 1: tR[min] 15.708; Area: 95.305%
Peak 2: tR[min] 23.308; Area: 4.695%

Table 2 entry 4 (Phosphate buffer, rt, 1 h 98% ee), (*R*)-**1**

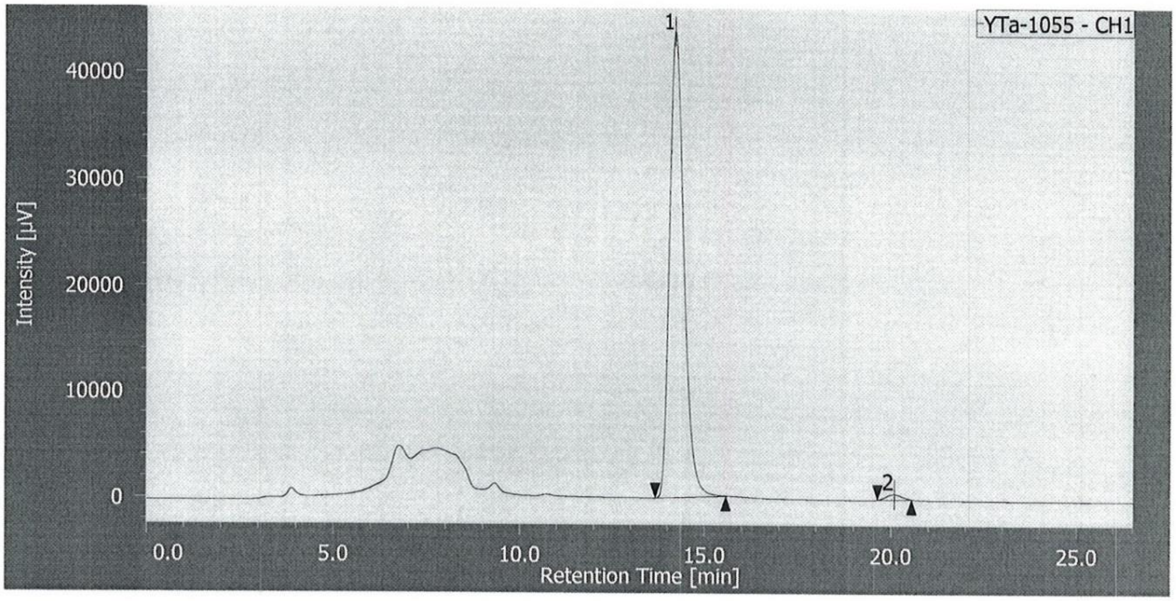

Peak 1: tR[min] 14.192; Area: 98.774%
Peak 2: tR[min] 20.092; Area: 1.226%

Table 2 entry 5 (Phosphate buffer, rt, 24 h 88% ee), (*R*)-**1**

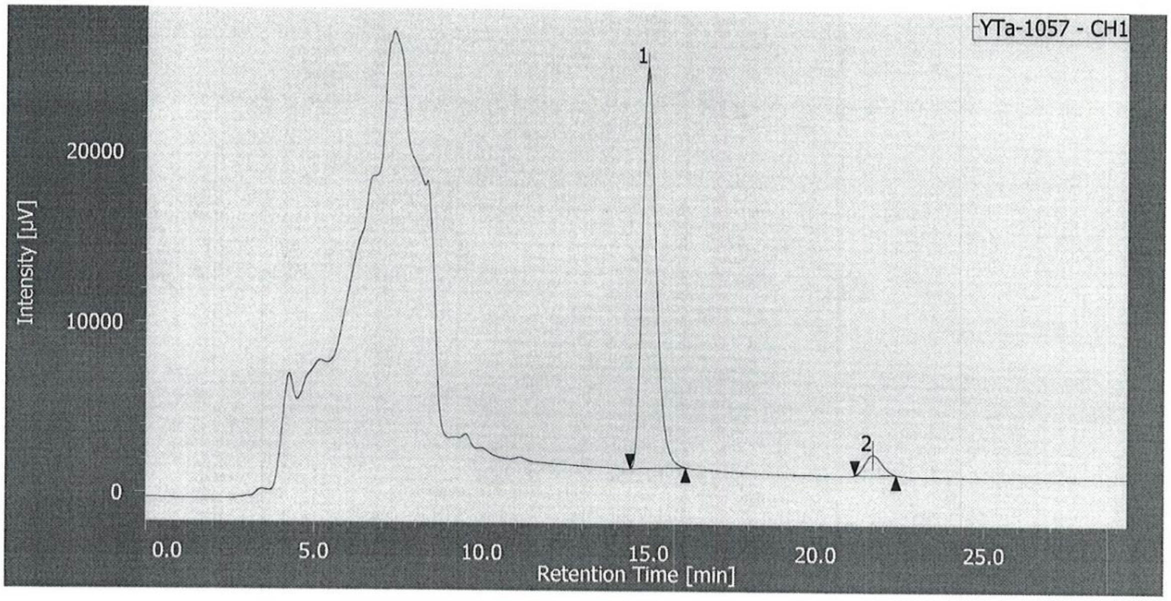

Peak 1: tR[min] 14.942; Area: 94.188%
Peak 2: tR[min] 21.675; Area: 5.812%

Table 2 entry 6 (Phosphate buffer, 37°C, 1 h 98% ee), (*R*)-**1**

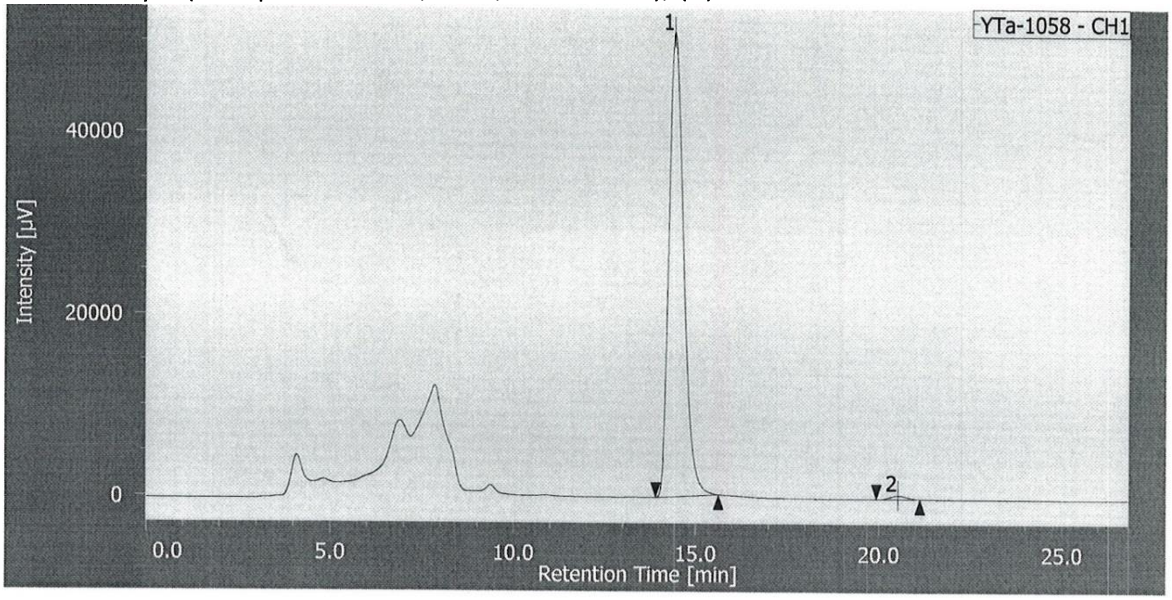

Peak 1: tR[min] 14.408; Area: 99.069%
Peak 2: tR[min] 20.508; Area: 0.931%

Table 2 entry 7 (Phosphate buffer, 37°C, 24 h 62% ee), (*R*)-**1**

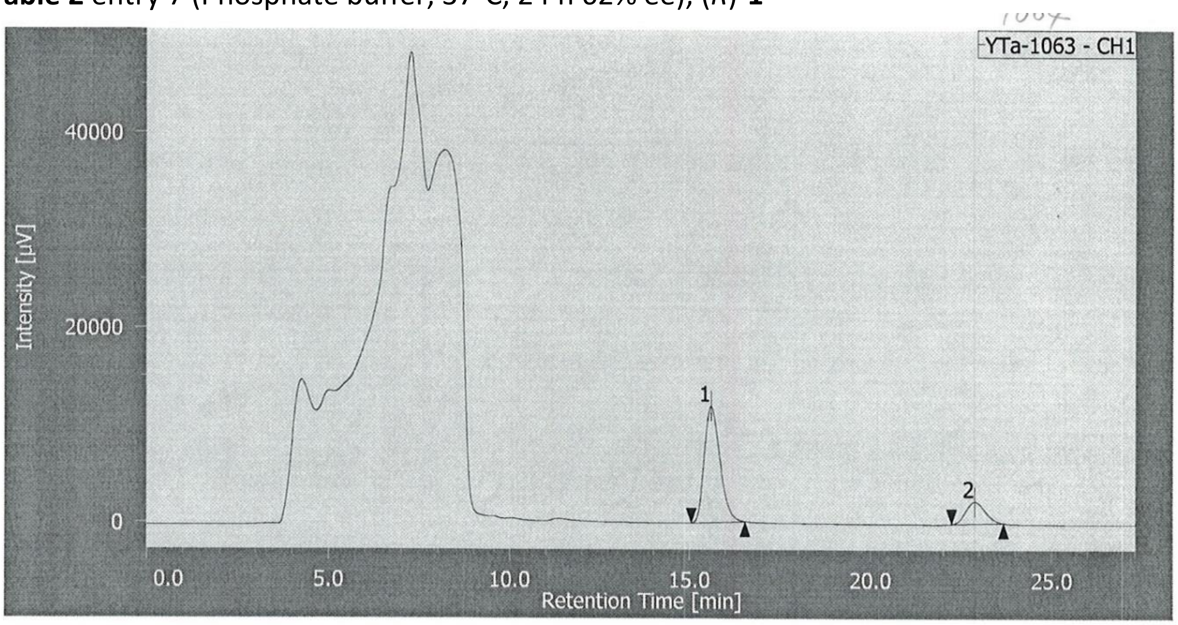

Peak 1: tR[min] 15.592; Area: 80.784%
Peak 2: tR[min] 22.867; Area: 19.216%

Table 2 entry 8 (water, rt, 1 h 89% ee), (*S*)-**1**

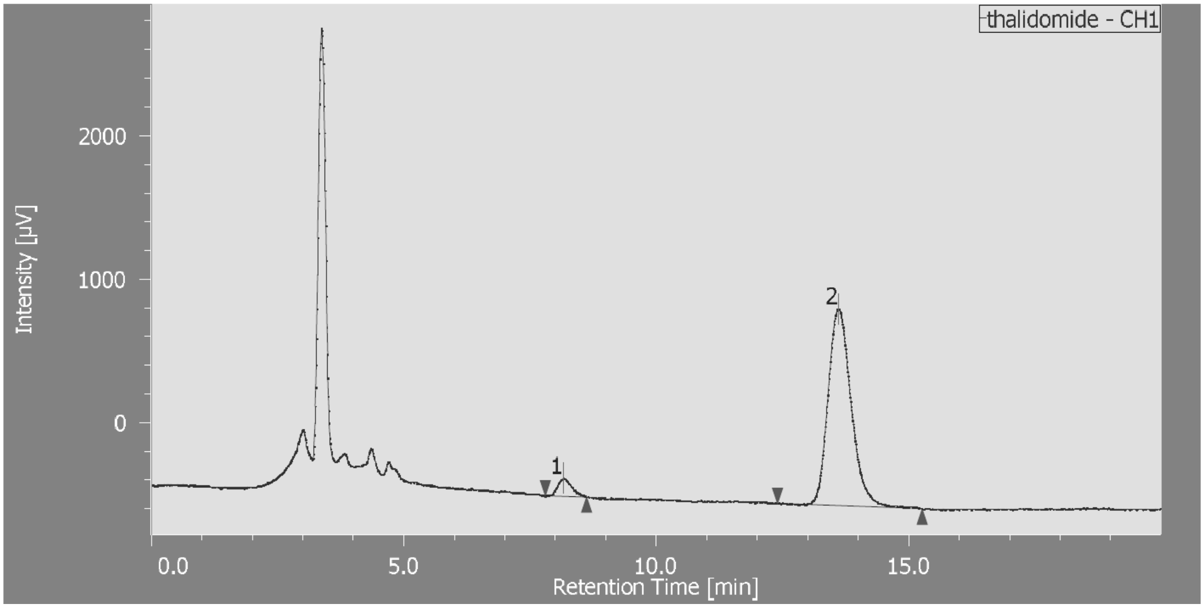

Peak 1: tR[min] 8.167; Area: 5.403%
Peak 2: tR[min] 13.600; Area: 94.597%

Table 2 entry 9 (water, rt, 1 h 87% ee), (*R*)-**1**

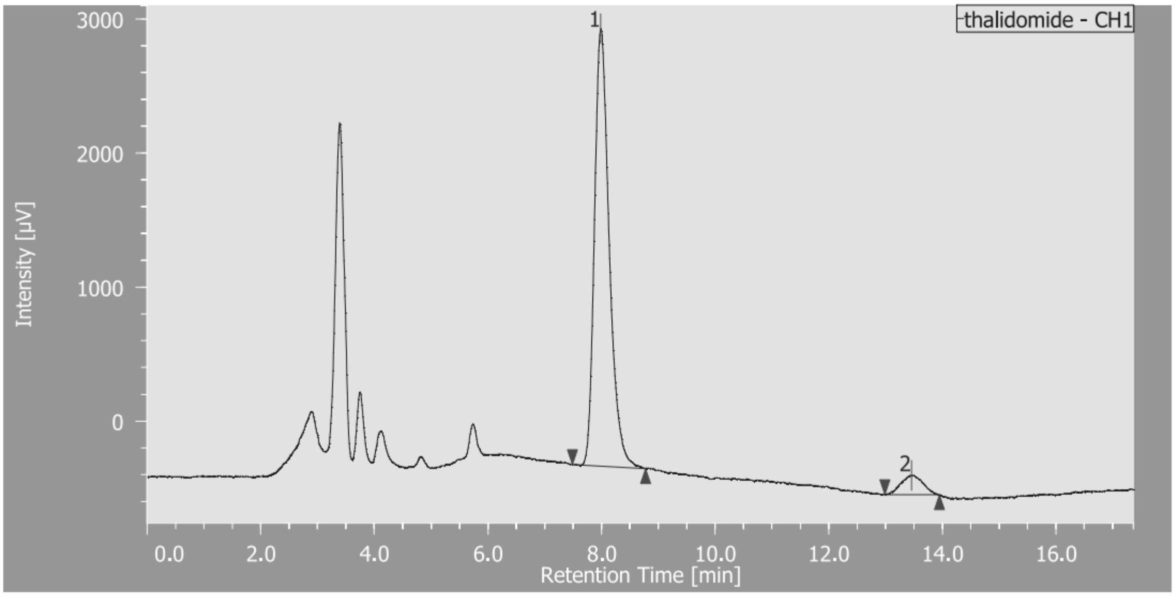

Peak 1: tR[min] 7.983; Area: 93.725%
Peak 2: tR[min] 13.450; Area: 6.275%

Table 2 entry 10 (water, rt, 1 h 74% ee), (*R*)-**1**

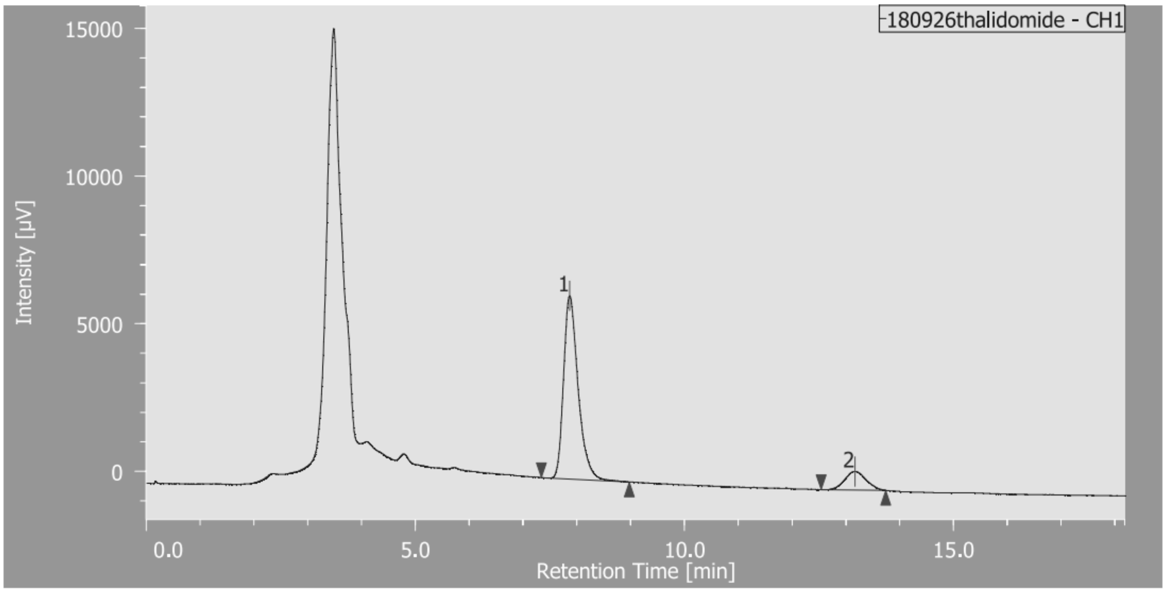

Peak 1: tR[min] 7.867; Area: 86.891%
Peak 2: tR[min] 13.167; Area: 13.109%

Table 2 entry 11 (water, rt, 1 h 61% ee), (*R*)-**1**

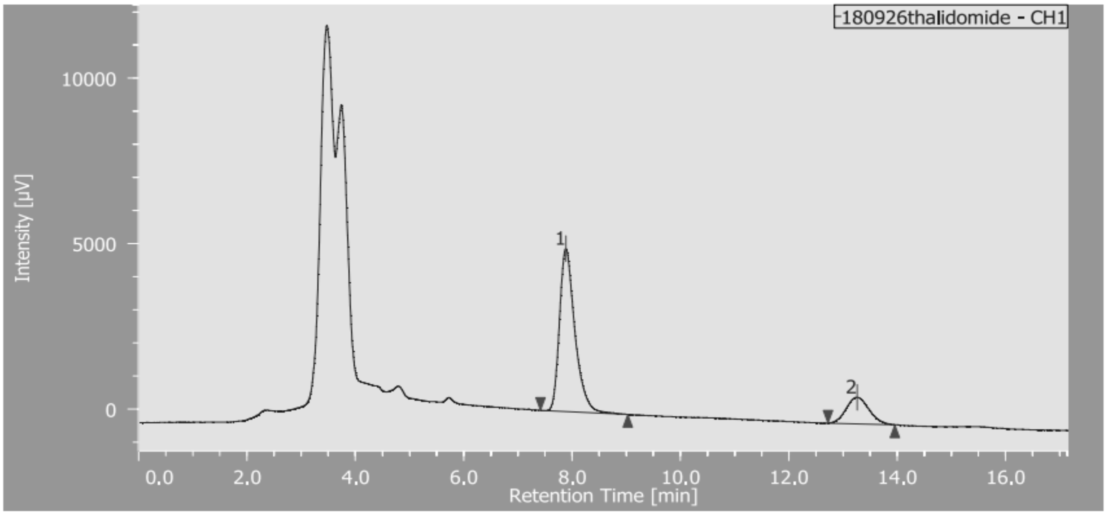

Peak 1: tR[min] 7.883; Area: 80.541%
Peak 2: tR[min] 13.258; Area: 19.459%

Fig. 4, 21% ee → 78% ee, 19% ee, (*R*)-**1**

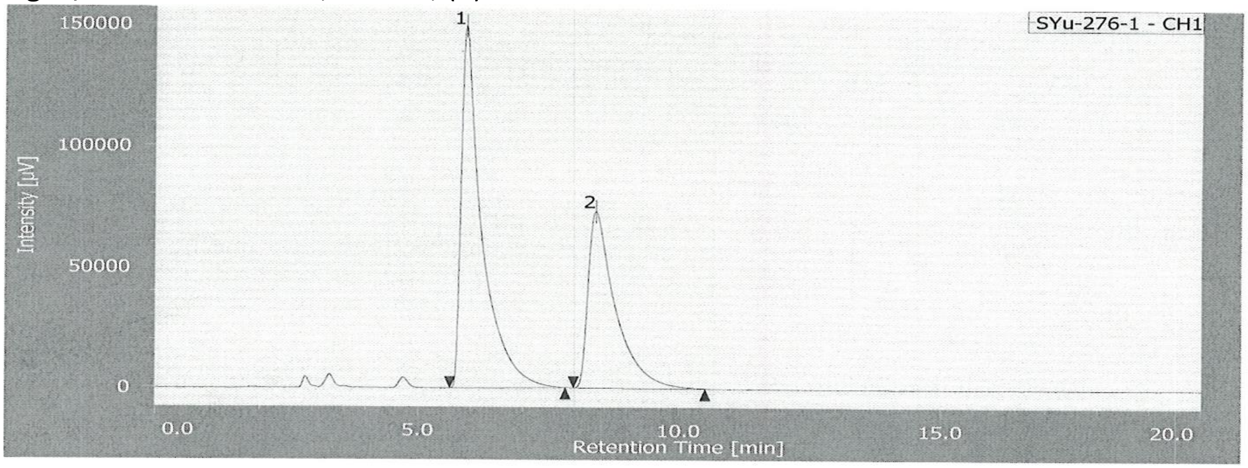

Peak 1: tR[min] 5.900; Area: 60.618%
Peak 2: tR[min] 8.392; Area: 39.382%

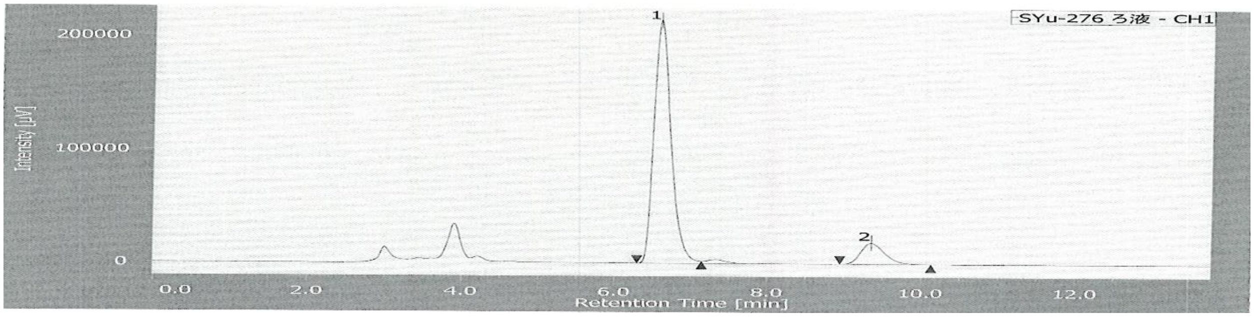

Peak 1: tR[min] 6.575; Area: 89.236%
Peak 2: tR[min] 9.350; Area: 10.764%

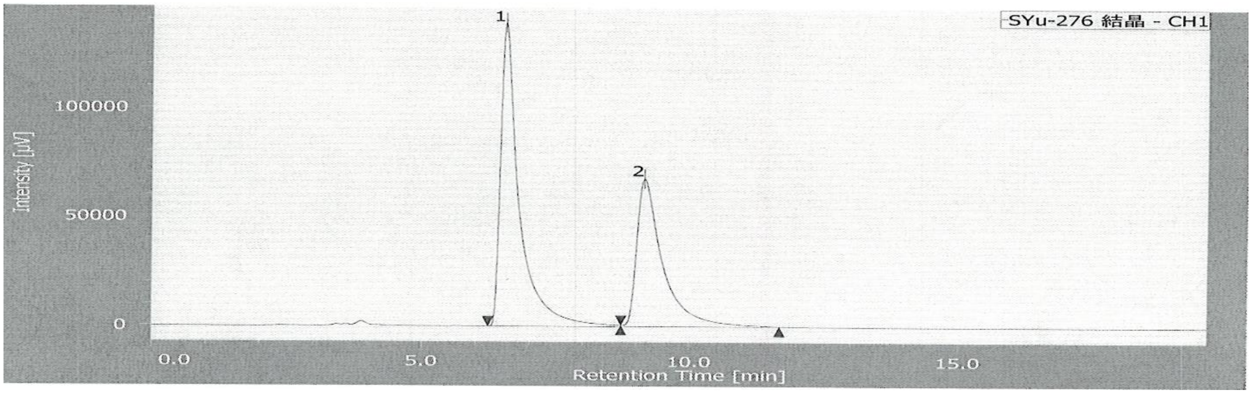

Peak 1: tR[min] 6.533; Area: 59.663%
Peak 2: tR[min] 9.142; Area: 40.337%

Fig. 4, 20% ee →74% ee, 16% ee, (*R*)-**1**

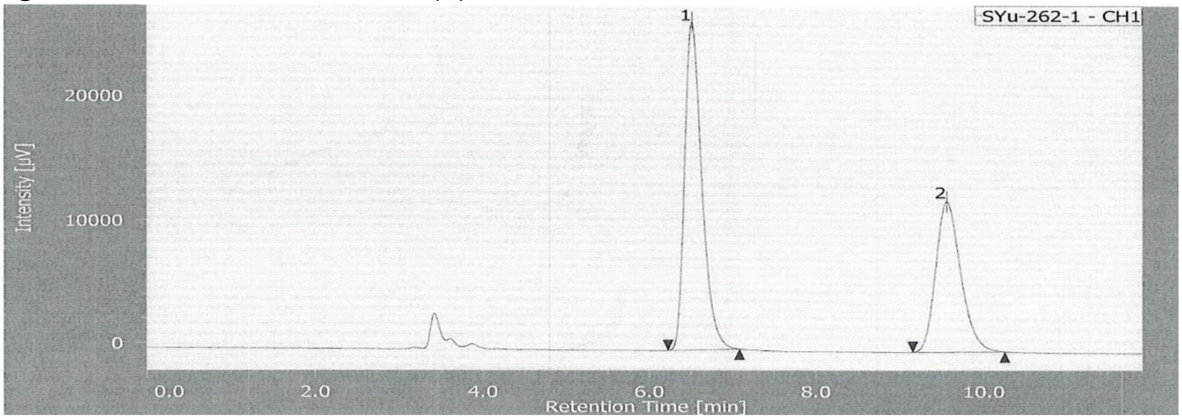

Peak 1: tR[min] 6.517; Area: 59.901%
Peak 2: tR[min] 9.558; Area: 40.099%

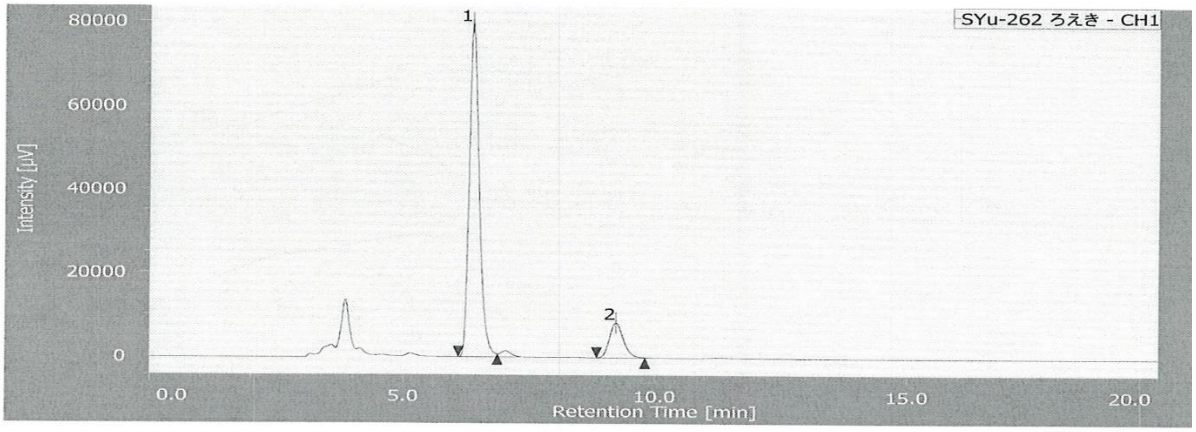

Peak 1: tR[min] 6.358; Area: 87.219%
Peak 2: tR[min] 9.225; Area: 12.781%

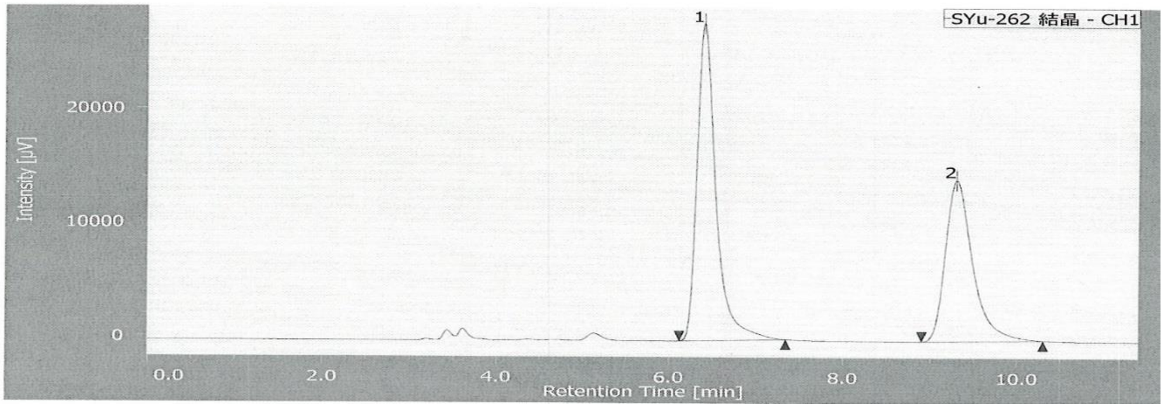

Peak 1: tR[min] 6.400; Area: 57.766%
Peak 2: tR[min] 9.300; Area: 42.234%

Fig. 4, 21% ee →72% ee, 20% ee, (*R*)-**1**

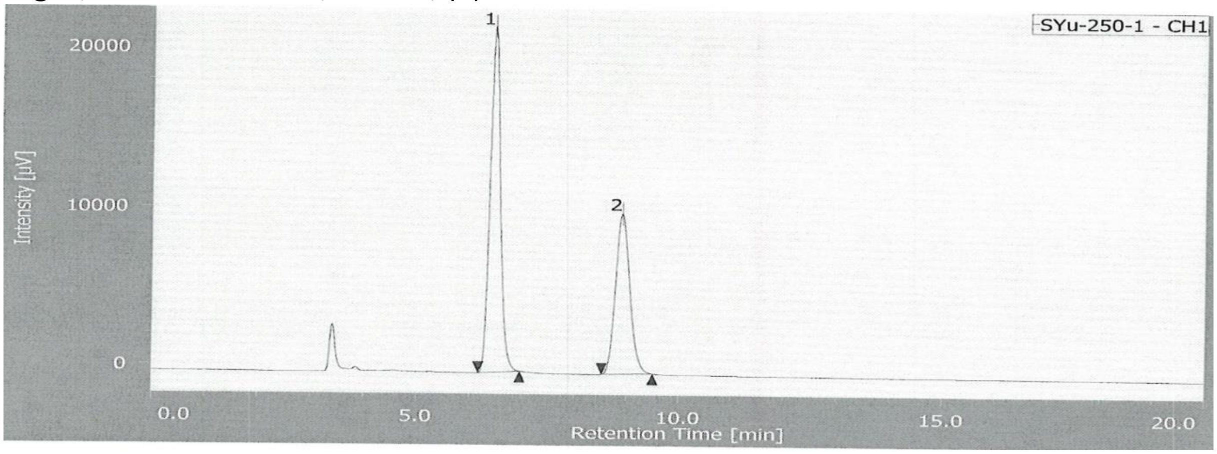

Peak 1: tR[min] 6.442; Area: 60.661%
Peak 2: tR[min] 8.900; Area: 39.339%

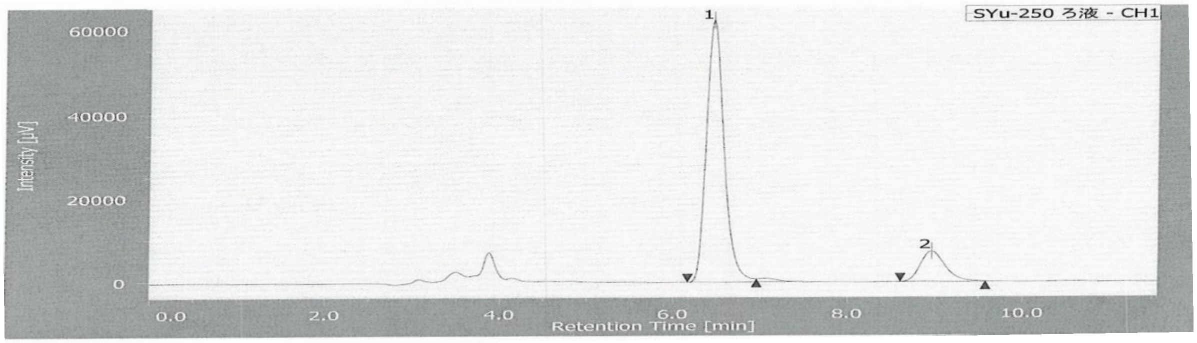

Peak 1: tR[min] 6.450; Area: 86.035%
Peak 2: tR[min] 8.958; Area: 13.965%

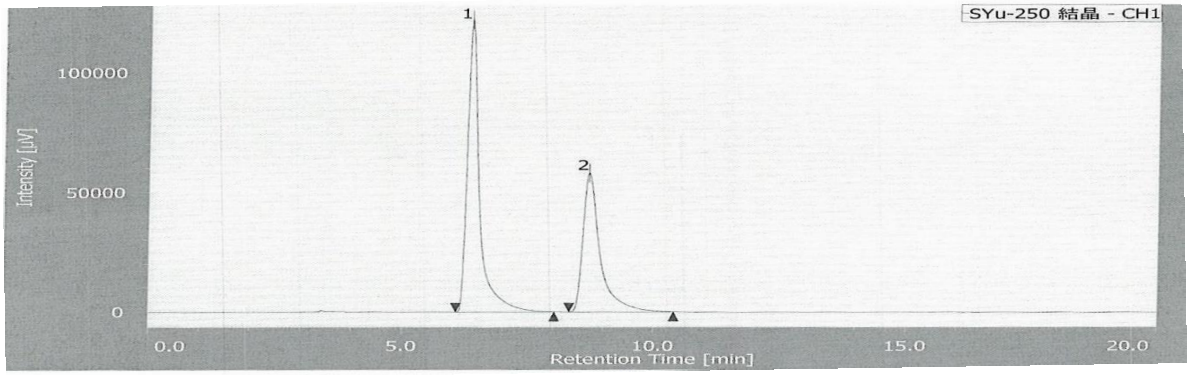

Peak 1: tR[min] 6.333; Area: 59.827%
Peak 2: tR[min] 8.692; Area: 40.173%

Fig. 7, 23% ee →76% ee, (*S*)-**2**

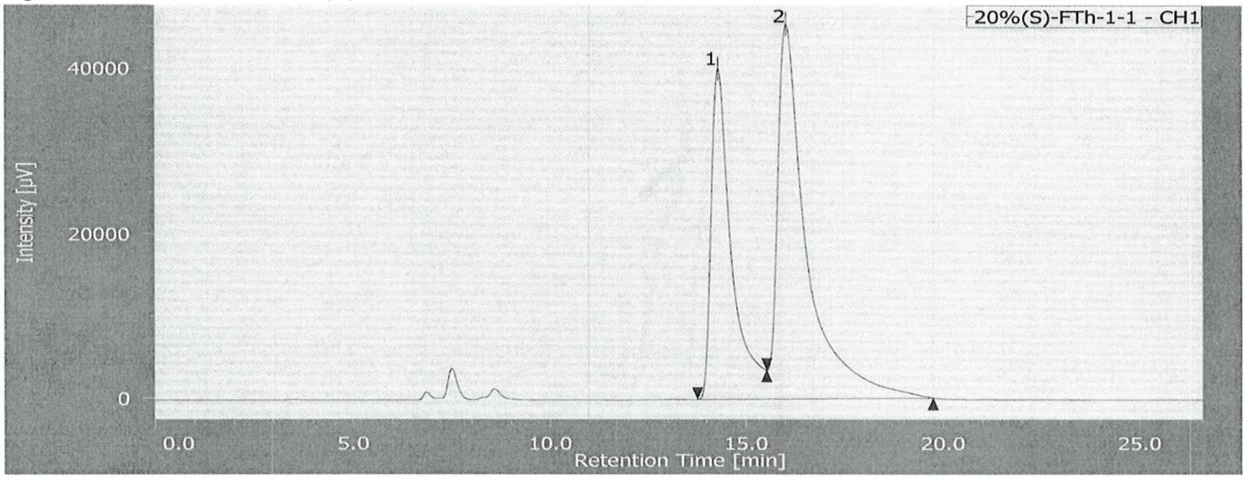

Peak 1: tR[min] 14.292; Area: 38.510%
Peak 2: tR[min] 16.025; Area: 61.490%

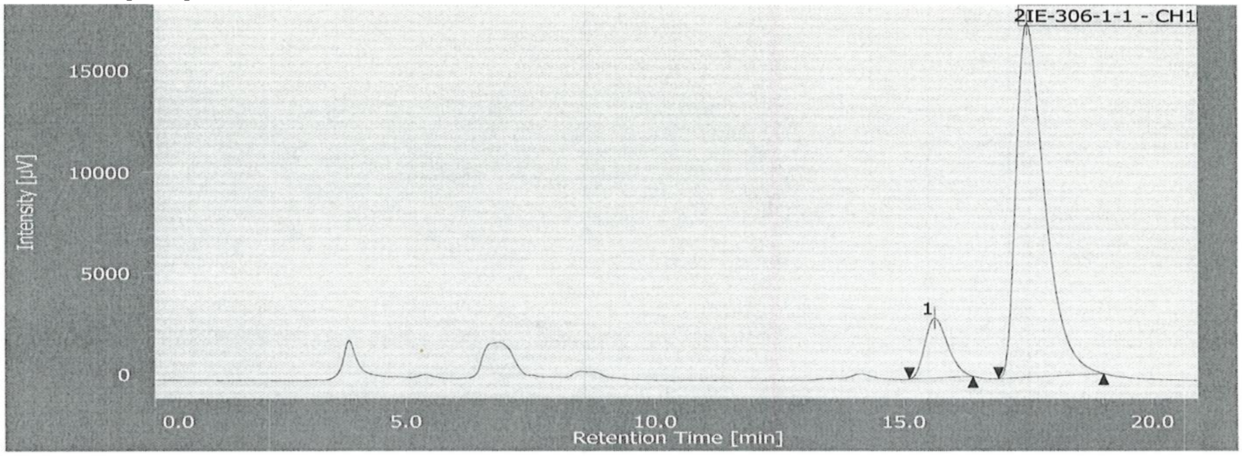

Peak 1: tR[min] 15.625; Area: 11.906%
Peak 2: tR[min] 17.475; Area: 88.094%
